# Longevity-associated BPIFB4 gene counteracts the inflammatory signaling

**DOI:** 10.1186/s12979-024-00424-5

**Published:** 2024-03-12

**Authors:** Monica Cattaneo, Andrea Baragetti, Alberto Malovini, Elena Ciaglia, Valentina Lopardo, Elena Olmastroni, Manuela Casula, Carolina Ciacci, Alberico L. Catapano, Annibale A. Puca

**Affiliations:** 1 Cardiovascular Department, IRCCS MultiMedica, Milan, Italy; 2 Department of Pharmacological and Biomolecular Sciences “Rodolfo Paoletti”, Università degli Studi di Milano, Milan, Italy; 3 Laboratory of Informatics and Systems Engineering for Clinical Research, Istituti Clinici Scientifici Maugeri IRCCS, Pavia, Italy; 4 Department of Medicine, Surgery and Dentistry, University of Salerno, Salerno, Italy; 5 Epidemiology and Preventive Pharmacology Service (SEFAP), Department of Pharmacological and Biomolecular Sciences, University of Milan, Milan, Italy

## Abstract

**Background:**

Increased levels of pro-inflammatory proteins in plasma can be detected in older individuals and associate with the so called chronic low-grade inflammation, which contributes to a faster progression of aged-related cardiovascular (CV) diseases, including frailty, neurodegeneration, gastro-intestinal diseases and disorders reflected by alterations in the composition of gut microbiota. However, successful genetic programme of long-living individuals alters the trajectory of the ageing process, by promoting an efficient immune response that can counterbalance deleterious effects of inflammation and the CV complications. This is the case of BPIFB4 gene in which, homozygosity for a four single-nucleotide polymorphism (SNP) haplotype, the Longevity-Associated Variant (LAV) correlates with prolonged health span and reduced risk of CV complications and inflammation. The relation between LAV-BPIFB4 and inflammation has been proven in different experimental models, here we hypothesized that also human homozygous carriers of LAV-BPIFB4 gene may experience a lower inflammatory burden as detected by plasma proteomics that could explain their favourable CV risk trajectory over time. Moreover, we explored the therapeutic effects of LAV-BPIFB4 in inflammatory disease and monolayer model of intestinal barrier.

**Results:**

We used high-throughput proteomic approach to explore the profiles of circulating proteins from 591 baseline participants selected from the PLIC cohort according to the BPIFB4 genotype to identify the signatures and differences of BPIFB4 genotypes useful for health and disease management. The observational analysis identified a panel of differentially expressed circulating proteins between the homozygous LAV-BPIFB4 carriers and the other alternative BPIFB4 genotypes highlighting in the latter ones a higher grade of immune-inflammatory markers. Moreover, in vitro studies performed on intestinal epithelial organs from inflammatory bowel disease (IBD) patients and monolayer model of intestinal barrier demonstrated the benefit of LAV-BPIFB4 treatment.

**Conclusions:**

Homozygosity for *LAV-BPIFB4* results in the attenuation of inflammation in PLIC cohort and IBD patients providing preliminary evidences for its therapeutic use in inflammatory disorders that need to be further characterized and confirmed by independent studies.

**Supplementary Information:**

The online version contains supplementary material available at 10.1186/s12979-024-00424-5.

## Introduction

Inflammation plays a central role in most age-related chronic disorders including cardiovascular (CV) and cerebrovascular diseases, the leading causes of death and disability worldwide [1]. Ageing process is accompanied by chronic low-grade inflammatory state, which is characterized by a constant background release of inflammatory mediators (including interleukins and cytokines), and by phenotypic changes of the haematopoietic cells, distinguished by somatic mutations leading to an aberrant proliferative potential known has been linked to not only well-known gastrointestinal inflammatory diseases, such as inflammatory bowel diseases (IBD), but also to cardiovascular risk factors [4, 5]to relate to CV disease [2].

Chronic low-grade inflammation is also the major feature of the gut microbiota dysregulation, also known as dysbiosis [3]. This condition has been linked to not only well-known gastrointestinal inflammatory diseases, such as inflammatory bowel diseases (IBD), but also to cardiovascular risk factors [4, 5].

Understanding the mechanisms that link inflammation and ageing could reveal new therapeutic targets and offer options to reduce the burden of CV morbidity in an ageing population. The comparison between genetic variants with a biological meaning and the circulating protein levels could provide insights into the underlying mechanisms. In this contest, circulating proteins could also help to understand the patho-physiology of age-related inflammatory diseases.

BPIFB4 is a blood circulating protein belonging to the BPI/LBP/PLUNC family proteins with a crucial role in the innate immune protection [6]. The homozygous for the minor allele of rs2070325 SNP in BPIFB4 associates with extraordinarily prolonged life-span in three geographical areas and, in linkage disequilibrium with other three SNPs, generates the Longevity Associated Variant (LAV) of BPIFB4 [6, 7]. The frequency of LAV homozygosity is 14% in long-living individuals and 10% in general population [6].

Homozygous carriers of LAV-BPIFB4 haplotype have higher circulating BPIFB4 levels, increased phosphorylated endothelial nitric oxide synthase in circulating mononuclear cells, lower atherosclerotic risk and preserved pericyte ensheathment of microvessels during aged cardiomyopathy [6, 8–10], suggesting that enhanced qualities and increased circulating quantities may account for the benefit of carrying the LAV-BPIFB4 gene. Likewise, delivery LAV-BPIFB4 in mouse model of human diseases improved revascularisation, reduced endothelial dysfunction, atherosclerosis and myocardial infarction-induced damage [6, 9–11]. Moreover, LAV-BPIFB4 exhibits rejuvenating effects on the immune and cardiac systems and vasculature accompanied with a deceleration in the progression of frailty [10, 12–14]. The benefits of LAV-BPIFB4 are linked to the potentiation of the proteostasis and ribosome biogenesis, [6, 10], and the resolution of inflammation through a mechanism that involves the interaction between SDF-1 and CXCR4, a receptor significantly expressed in the myeloid lineage and in macrophages, which promotes the M2 (antinflammatory) skewing of macrophages [9, 15].

Here, we performed an observational study in human PLIC cohort stratified for the homozygous LAV-BPIFB4 genotype.

We describe the measurement, processing and downstream analysis of 368 blood plasma proteins evaluated across the BPIFB4-genotyped PLIC participants using the antibody-based proximity extension assay (PEA). In addition, in order to evaluate the therapeutic effects in inflammatory disease, we tested the forced LAV-BPIFB4 expression on in vitro human IBD biopsies and monolayer model of intestinal barrier and reported the potential benefits of the treatment.

## Materials and methods

### Study population

We included 591 individuals from the PLIC study, a population-based cohort representative of the general population of the northern area of Milan (“PLIC”, “Progressione delle Lesioni Intimali Carotidee”) [16]. PLIC study is an ongoing single-centre, observational, cross-sectional, and longitudinal study of subjects enrolled on a voluntary basis in 1998 to 2000 and followed up for about 20 years (to date a total of 6 visits, on average every 4 years [17]. The study is conducted by the Center for the Study of Atherosclerosis at the E. Bassini Hospital (Cinisello Balsamo, Milan, Italy) with the coordination of the Epidemiology and Preventive Pharmacology Centre (SEFAP) of the Università degli Studi di Milano (Milan, Italy).

The flow chart shown in Supplementary Fig. 1 explains the methodology used to select a part of total PLIC participants.

The study was approved by the Ethics Committee of the University of Study of Milan (approved on 06-02-2001 SEFAP protocol n°0003/2001). Informed consent was obtained from subjects (all over 18 years-old), in accordance with the Declaration of Helsinki.

### TaqMan SNP genotyping assay

DNA was extracted from peripheral blood of healthy subjects (QIAamp DNA blood midi kit, Qiagen, Düsseldorf, Germany) and was genotyped using Taqman probe rs2070325.

### Measurement of the plasmatic expression of the proteomics panel

The expression of 368 proteins were analysed by Proximity Extension Assay (PEA) using Cardiovascular II, Cardiovascular III, Cardiometabolic, and Inflammation panels of the OlinkTM platform, as previously described [16, 18]. A total number of 10/368 proteins were present on more than one panel, with 358 unique proteins analysed.

Data are expressed as Normalized Protein eXpression (NPX) values.

### Statistical analysis of proteins expression data

The two-sided Wilcoxon rank sum test was applied to test the null hypothesis of no difference in terms of proteins expression between the homozygous for the rs207035 minor allele (GG), and the homozygous and heterozygous for rs207035 major allele (AA/AG). Non-parametric statistical tests were applied since most proteins’ distribution deviated significantly from the normality assumptions in at least one of the two genotypes groups (Shapiro-Wilk test of normality p-value < 0.05). The median of the difference between BPIFB4_rs2070325_(GG) and BPIFB4_rs2070325_(AA/AG) genotypes and corresponding 95% confidence interval has been computed to estimate the difference in location parameters for each protein as provided by the *wilcox.test* function implemented in the R package called *stats*. The Benjamini-Hochberg (BH) correction has been applied to control the false discovery rate: BH adjusted p-values < 0.05 have been considered evidence of statistically significant differences in terms of proteins expression between genotypes.

The Spearman correlation coefficient has been computed to estimate the degree of correlation between proteins analysed in two different panels. Proteins showing Spearman *r* < 0.8 and/or showing evidence of differential expression (p-value < 0.05) in only one out of the two panels have been excluded from the analysis. In the remaining cases one of the two duplicated proteins has been randomly selected to be included in the analysis. Quantile – quantile plots of observed vs. expected -log10 BH unadjusted p-values and λ value have been obtained by the *qqPlot* (*GWASTools* package) and *P_lambda* (*QCEWAS* package) functions.

The quantile regression was applied by the *rq* function implemented in the R package called *quantreg* to estimate the difference in terms of median value of each protein’s distribution between BPIFB4_rs2070325_(GG) and BPIFB4_rs2070325_(AA/AG) genotypes with/without adjustment for covariates. A bootstrap approach (*n* = 10,000 bootstrap samplings) has been applied to estimate standard error and p-value.

Missing values for the LDL-C variable (*n* = 5 subjects with missing values) have been imputed by the median value of the corresponding variable’s distribution before performing the quantile regression analyses.

### Gene ontology enrichment analysis

Proteins have been mapped to the corresponding Entrez gene ID by the *bitr* function implemented in the R package called *clusterProfiler* or by manual mapping. Gene Ontology (GO) analyses have been performed exploring molecular function (MF), biological process (BP) and cellular components (CC) aspects by the *enrichGO* function implemented in the R package called *clusterProfiler*. To this aim, the set of genes reaching BH unadjusted p-value < 0.05 from univariate tests comparing proteins expression between genotypes (thus defining the set of genes of interest) and the complete set of analysed proteins passing quality control and with a corresponding Entrez ID (background genes) were used as input. The minimal size of genes annotated by ontology term for testing (*minGSSize*) was set to 10, while no limit was set to the maximal size of genes annotated for testing (*maxGSSize*). BH adjusted p-values < 0.05 have been considered evidence of statistically significant enrichment of a specific gene ontology category.

Statistical and gene ontology analyses have been performed by functions implemented in the R software environment for statistical computing and graphics version 4.3.1 (www.r-project.org).

### Cell maintenance, treatment and transfection

Human colon cancer cells (Caco-2) were purchased from ATCC (Tell City, USA) and maintained in RPMI (Thermo Fisher Scientific, Waltham, USA) supplemented with 10% foetal bovine serum (FBS) (Thermo Fisher Scientific).

Cells were transfected with the indicated plasmids using the TransiT-X2 reagent (Mirus, Madison, USA) according to the manufacturer’s instructions. After 48 h, butyrate (2 mM, Sigma-Aldrich, United Kingdom) was added at indicted time.

### Animal model and primary murine splenocytes isolation

We used *n* = 4 C57BL/6 mice from Jackson Laboratories. The Institutional Animal Care Use Committee of Neuromed Medical Center approved all animal experiments [n° 327/2023-PR]. Mouse colonies were maintained in the animal facility at IRCCS Neuromed, Pozzilli (IS), Italy. Ten-week-old male C57BL/6 mice were anesthetized with 5% isoflurane in 100% O2 (delivery rate, 5 L/min), and placed in dorsal recumbent position on a homoeothermic blanket (N-HB101-S-402) to maintain body temperature at 37 °C. Anaesthesia was maintained with 1% isoflurane in 100% O2 at 1.5 L/min, administered by means of a facemask connected to a coaxial circuit (Fluovac anaesthetic mask), and eutanized by beheading to collect tissue samples. In details, to isolate murine splenocytes, spleen was scalpel-cut and maintained into tubes containing RPMI-1640 (Gibco®, ThermoFisher Scientific) supplemented with 10% (v/v) fetal serum bovine (FBS, Gibco®, ThermoFisher Scientific) and 2% (v/v) penicillin-streptomycin (Aurogene). Then, spleen was placed on a 70 μm cell strainer previously held on a 50mL tube. RPMI-1640-supplemented was added and the spleen was pressed with the plunger of a 5 mL syringe. Then, RPMI-1640-supplemented were added to clean the filter. The cell suspension was centrifuged at 400xg for 5 min. Splenocytes were plated in 96-well plate and treated with LPS 1 µg/mL for 18 h in presence or absence of recombinant LAV-BPIFB4 (18ng/mL). Further, supernatants were collected for TNFSF14 secretion dosage.

### Enzyme-linked immunosorbent assay (ELISA)

TNFSF14 level was determined using Mouse Tumor Necrosis Factor Ligand Superfamily, Member 14 (TNFSF14) ELISA kit (antibodies; Cat. No. A5014) following the manufacturer’s protocol. Briefly, supernatants were incubated for 2 h at 37 °C in the assay coated microplate. After removing any unbound substances, Reagent A was added to the wells and incubated for 1 h at 37 °C. After washing, Reagent B was added to the wells and incubated for 1 h at 37 °C. Following several washing steps, substrate solution was added, and the consequent color development was stopped. Optical density was measured at 450 nm.

### RNA extraction and quantitative real-time analysis

RNA was extracted with RNeasy (Qiagen, Germantown, USA), following the protocol provided by the manufacturer. Total RNA concentration and quality were determined using a Nanodrop spectrophotometer (Nanodrop 1000, Thermo Fisher Scientific). Before retro-transcription, DNAse I (Thermo Fisher Scientific) was used to remove genomic DNA contamination. Subsequently, Superscript III, Oligo(dT)12–18, dNTPs mix, and RNaseOUT (Thermo Fisher Scientific) were used to synthesize cDNA, following the manufacturer’s protocol. QuantStudio™ 6 Flex Real-Time PCR System (Applied Biosystems) and SYBR Green PCR Master Mix (Applied Biosystems, Life Technology) were employed to conduct Real-Time-qPCR analyses, on triplicate samples of retrotranscribed cDNA. Expression levels were normalized to 18 S. Data were expressed as 2-(ΔΔCt). Primer sequences are the following:

- BPIFB4-F: GTGGGTGTCTACCTGAGCTTGT
- BPIFB4-R: GCTCAATGACCAGCCGAGGATA
- 18S-F: CTCATGGAGGTGCTGGTG
- BPIFB4-F: GTGGGTGTCTACCTGAGCTTGT

### Western blotting

Cells were lysed in RIPA buffer containing protease and phosphatase inhibitor cocktail (Sigma-Aldrich). Protein concentration was determined using the Bradford assay (Sigma-Aldrich). Total proteins were separated by electrophoresis using 4–12% NuPAGE Bis-Tris protein gels (Thermo Fisher Scientific), transferred onto a polyvinylidene difluoride (PVDF) membrane (GE Healthcare, Buckinghamshire, UK), and probed overnight at 4 °C with the following primary antibodies: anti-BPIFB4 (dilution 1:1000, Clinsciences, Guidonia Montecelio, Italy, custom made), anti-H3 (dilution 1:1000, Abcam, Cambridge, UK), anti H3k9ac (dilution 1:1000, Millipore, Darmstadt, Germany), anti-Vinculin (dilution 1:5000, Cell Signaling Technology, Massachusetts, United States) and anti-GAPDH (dilution 1:5000, Cell Signaling Technology). Secondary IgG HP-conjugated anti-rabbit HRP-linked antibody (dilution 1:3000, Cell Signaling Technology) was applied for 1 h at room temperature. Blots were revealed by Western Chemiluminescent Substrate Westar Ultra 2 (Cyanagen, Bologna, Italy) using UVITEC Alliance Q9 (UVITEC, Cambridge, UK). Densitometric quantifications were normalized relative to housekeeping signal using UVITEC software.

### IBD biopsies cultured in vitro

Tissue samples from IBD patients were collected (*N* = 6). The study was approved by the Local Ethics Committee (Comitato Etico Campania Sud protocol prot./SCCE n.11,174 21/01/2019). Informed consent was obtained from subjects, in accordance with the Declaration of Helsinki. Fragments were placed in culture and challenged in presence/absence of the recombinant LAV-BPIFB4 protein and LPS. After 3 h, fragments were harvested, washed and freezed in OCT. Conditioned media were assayed for the release of multiple cito-chemokines that are considered reliable inflammation markers.

### Cito-chemokines detection

IL-8, IL-6 and MCP-1 levels in conditioned media of IBD tissues were determined using a beads-based multiplex ELISA (LEGENDplexTM, Biolegend, USA). Medium was incubated for 2 h with the beads and for 1 h with the detection antibodies, followed by 30 min incubation with SA-PE. After washing, beads were resuspended in washing buffer and acquired using a FACS VERSE flow cytometer (BD Biosciences). Data were analyzed with the LEGENDplex Data Analysis Software.

### Workflow diagram

**Fig. 1:**
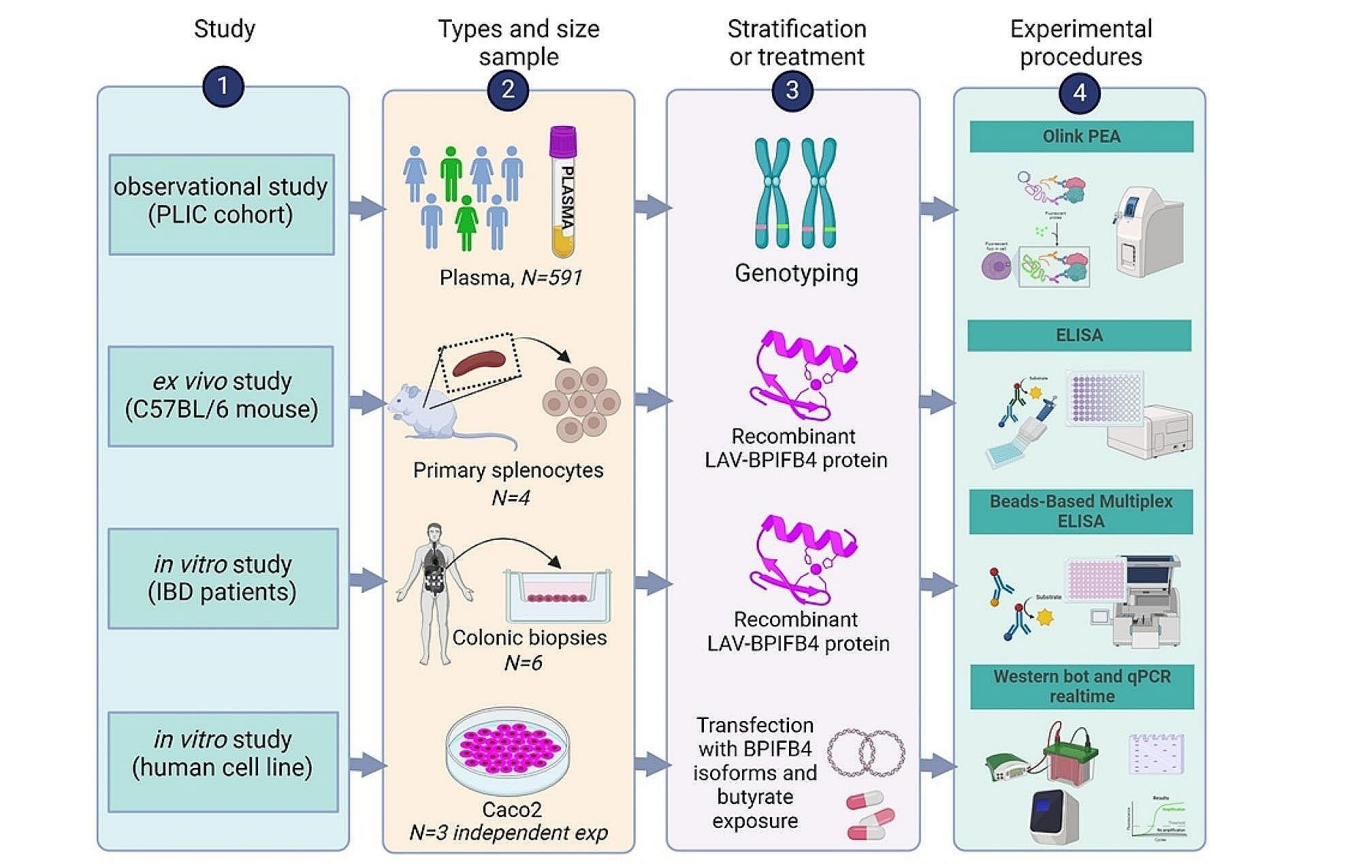
Work flow diagram. Workflow diagram summarizing the multiple types of study designs including the specimen type, number of samples, number of replicates, the treatments and the experimental procedures

The workflow diagram shown in Fig. 1 summarizes the multiple types of study designs.

## Results

### Homozygosity for *LAV-BPIFB4* results into reduced expression of plasmatic markers related to inflammation

We conducted proteomic profiling on blood plasma samples collected from 591 PLIC participants using PEA. A total of 358 protein analytes were measured according to the BPIFB4 genotype, comparing individuals homozygous for the rs207035 minor allele (GG, *n* = 89, 15.1%), named “homozygous LAV-BPIFB4*”* and individuals homozygous and heterozygous for rs207035 major allele (AA and AG, n = 502, 84.9%) named “others”. Epidemiologic and clinical data of the two subgroups are reported in Table 1. A significantly lower frequency of subjects of female sex and higher LDL-C concentrations were observed in homozygous LAV-BPIFB4 compared to other genotypes carriers (p-value < 0.05). No statistically significant differences in terms of the remaining variables distribution were observed between genotypes (p-value > 0.05).

**Table 1:** Distribution of variables in the two subgroups of PLIC participants classified according to the BPIFB4 genotype

		BPIFB4 Genotype		
Variables	Others		Homozygous LAV-BPIFB4	p-value
N	**502**		**89**	
Sex (Female), %	69.92		56.18	**0.01**
Age, years	54.66 (8.37)		54.36 (7.29)	0.75
BMI	26.65 (4.29)		26.30 (3.77)	0.47
WHR	0.87 (0.74)		0.82 (0.05)	0.24
SBP, mm Hg	131.27 (15.48)		130.10 (17.68)	0.56
DBP, mm Hg	82.53 (8.66)		83.15 (9.98)	0.55
Smoke: former, %	24.7		29.21	0.49
Smoke: current, %	17.93		20.22	
Total Cholesterol	220.59 (38.24)		227.78 (40.91)	0.11
HDL-C	56.89 (14.95)		54.81 (15)	0.23
TG**	89.5 [63–130]		90 [68–126]	0.66
LDL-C	142.5 (35.88)		151.63 (37.7)	**0.03**
apoA1	151.1 (24.8)		148.59 (23.88)	0.43
apoB	112.09 (24.33)		116.85 (26.83)	0.13
Glucose	89.63 (13.04)		90.24 (10.61)	0.63
Antihypertensive trt, %	20.92		25.84	0.3
Antidiabetic trt, %	1		0	0.34
Lipid-lowering trt, %	6.77		7.87	0.71
Antithrombotic trt, %	2.19		0	0.16
Mean IMT	0.62 (0.12)		0.64 (0.12)	0.1
Plaque, %	0		0	-
CHD, %	0		0	-
SCORE < 2, %	72.26		70.79	0.76
SCORE 2–5, %	24.75		24.72	
SCORE > = 5, %	2.99		4.49	
Metabolic syndrome %	21.31		21.69	0.94

Characteristics are presented as means and standard deviations (SD) for normally distributed variables, median and interquartile ranges [IQR] for non-normally distributed variables, or percentages for dichotomous variables. Categorical variables were compared by chi-square tests. Variables with normal distribution were compared using t-test, while variables with skewed distribution were compared using Mann-Whitney.

**Fig. 2:**
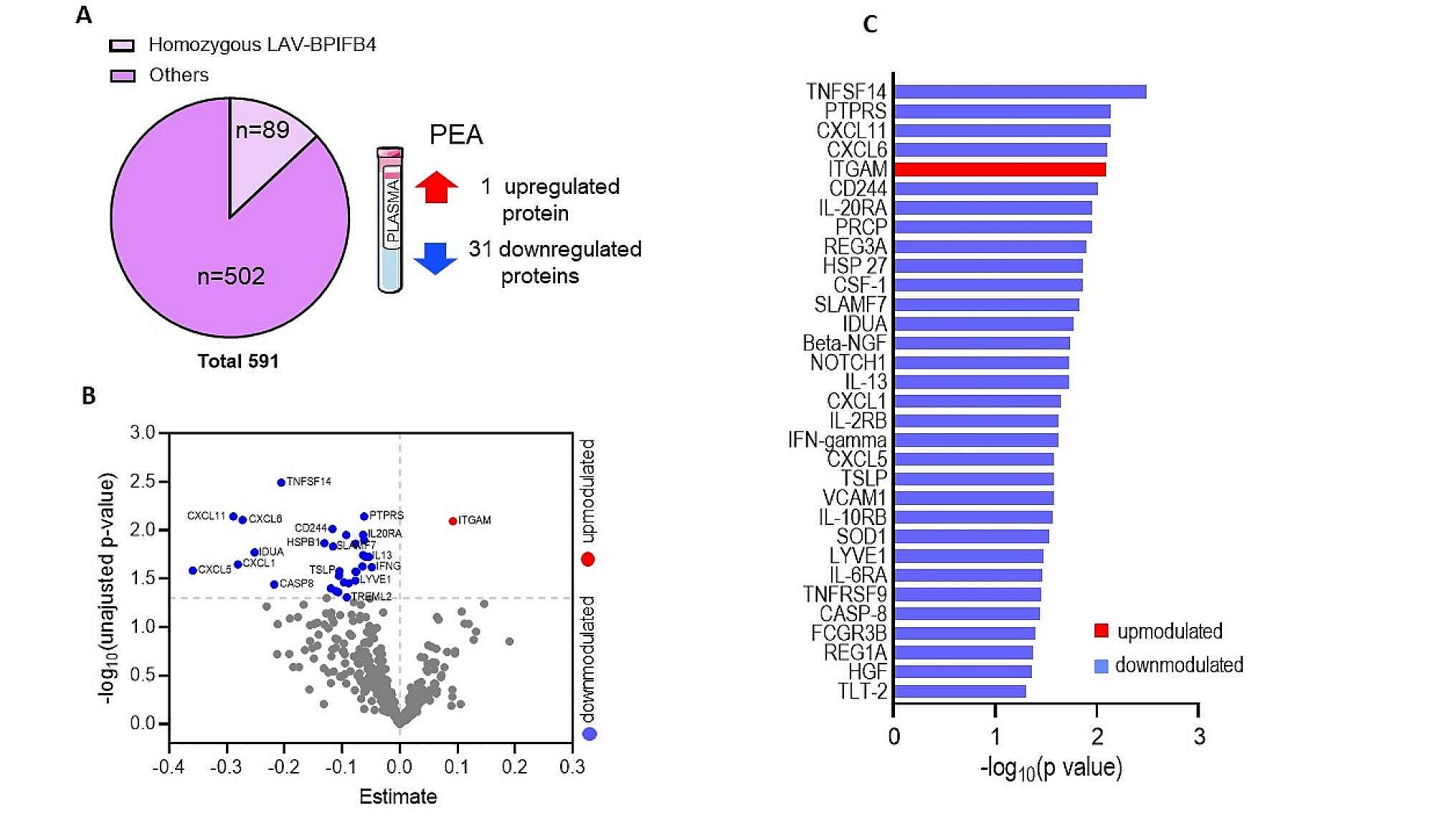
Proximity extension assay performed on plasma derived from PLIC cohort. **A, B)** Experimental design and volcano plot showing differentially circulating protein expression between homozygous LAV-BPIFB4 carriers (*n* = 89) and others (*n* = 502). **B)** Volcano plot, Estimate, median of the difference between homozygous LAV-BPIFB4 carriers and others; -log10 unadjusted p-value = -log 10 p-value from Wilcoxon rank sum test. Proteins showing higher and lower values in homozygous LAV-BPIFB4 carriers compared to others, reaching unadjusted p-value < 0.05, are represented in red and blue, respectively. Proteins showing no evidence of differences in terms of distribution between genotypes (unadjusted p-value ≥ 0.05) are depicted in grey. **C)** Bar graph indicating all differentially secreted proteins, -log10 unadjusted p-value, proteins showing higher and lower values in homozygous LAV-BPIFB4 carriers compared to others, reaching unadjusted p-value < 0.05, are represented in red and blue, respectively

In total, 32 differentially expressed proteins were identified between the two groups with a BH unadjusted p-value < 0.05 (Fig. 2; Table 2 and Supplementary Table 1), however, the difference was not statistically significant after false discovery rate correction (BH adjusted p-value ≥ 0.05, Supplementary Fig. 2). Of these, 31 proteins were downmodulated and only one upregulated in the homozygous-LAV group. The latter, ITGAM, is the alpha subunit of integrin-β2 which plays a crucial role in limiting unwanted inflammatory immune responses and de-regulating proinflammatory processes including Toll-Like Receptors (TLR) pathway [19]. Notably, non-synonymous coding variant of the ITGAM gene is a risk factor to systemic lupus erythematosus (SLE) [20]. Conversely, the majority of the down-modulated proteins included several cytokines and chemokines belonging to pro-inflammatory pathways. Among the top downmodulated proteins, TNFSF14 is a ligand for a member of the tumour necrosis factor receptor superfamily predominantly expressed in the spleen and implicated in the herpesvirus entry into cells during infection [21]. Quantile regression showed that the median value of the TNFSF14 distribution was significantly lower in homozygous LAV-BPIFB4 compared to other genotypes carriers also after adjustment for sex and/or LDL-C (Beta < 0, p-value < 0.05, Supplementary Table 2), being variables showing evidence of unbalanced distribution between genotype groups (Table 1).

**Fig. 3:**
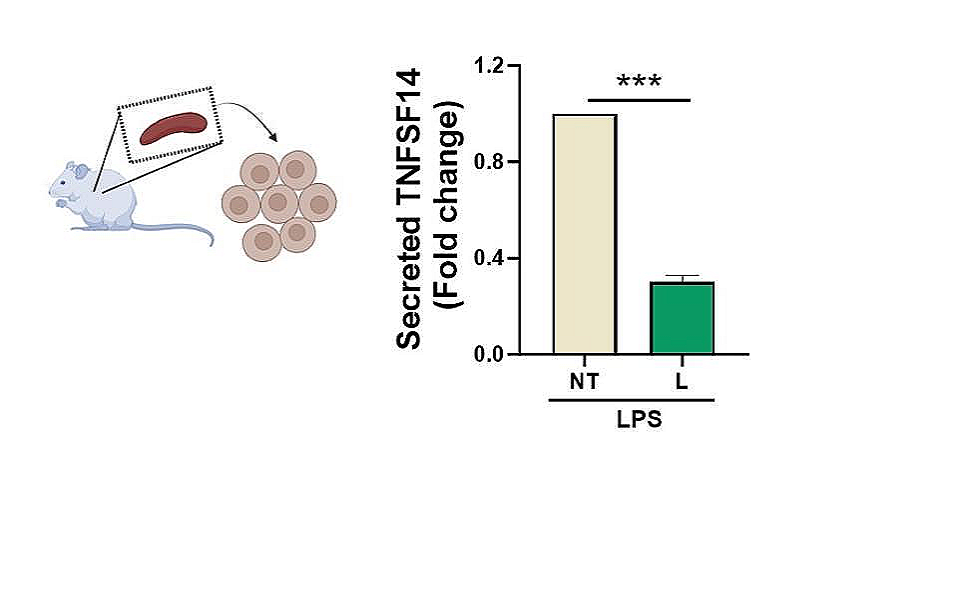
TNFSF14 secretory levels in LPS-treated murine splenocytes with or without recombinant LAV-BPIFB4 protein. Histogram shows TNFSF14 secretion levels (pg/mL) in supernatants from LPS (1µg/mL)-treated splenocytes in absence (NT) or presence of recombinant LAV-BPIFB4 (18ng/mL) (L) for 18 h from C57BL/6 mice’ spleens detected by ELISA. Data were analyzed using unpaired two-tailed t-test (*n* = 4)

In the view to corroborating the pattern profile of TNFSF14 and taking into account the tissue specificity, we analysed the levels of the released protein in the supernatants of primary murine splenocytes exposed to recombinant LAV-BPIFB4 protein. Similarly, LAV-BPIFB4 conditioning of LPS-treated splenocytes significantly reduced the amount of secreted TNFSF14 (Fig. 3), strengthening the depressed regulation evoked by LAV haplotype in both immune and splenic compartments.

**Table 2:** Set of selected proteins

					p-value		
**Protein symbol**	**Uniprot ID**	**Estimate**	**L95**	**U95**	**BH unadjusted**	**BH adjusted**	**Lower in**:
TNFSF14	O43557	-0.206	-0.339	-0.070	0.0032	0.4125	Homo-LAV-BPIFB4 carriers
PTPRS	Q13332	-0.062	-0.106	-0.017	0.0072	0.4125	Homo-LAV-BPIFB4 carriers
CXCL11	O14625	-0.289	-0.494	-0.083	0.0072	0.4125	Homo-LAV-BPIFB4 carriers
CXCL6	P80162	-0.273	-0.481	-0.072	0.0078	0.4125	Homo-LAV-BPIFB4 carriers
ITGAM	P11215	0.092	0.025	0.159	0.0081	0.4125	Others
CD244	Q9BZW8	-0.117	-0.202	-0.030	0.0097	0.4125	Homo-LAV-BPIFB4 carriers
IL-20RA	Q9UHF4	-0.064	-0.112	-0.015	0.0111	0.4125	Homo-LAV-BPIFB4 carriers
PRCP	P42785	-0.093	-0.166	-0.022	0.0112	0.4125	Homo-LAV-BPIFB4 carriers
REG3A	Q06141	-0.062	-0.110	-0.013	0.0127	0.4125	Homo-LAV-BPIFB4 carriers
HSP 27	P04792	-0.131	-0.229	-0.027	0.0136	0.4125	Homo-LAV-BPIFB4 carriers
CSF-1	P09603	-0.077	-0.135	-0.016	0.0137	0.4125	Homo-LAV-BPIFB4 carriers
SLAMF7	Q9NQ25	-0.116	-0.209	-0.022	0.0147	0.4125	Homo-LAV-BPIFB4 carriers
IDUA	P35475	-0.252	-0.455	-0.046	0.0170	0.4125	Homo-LAV-BPIFB4 carriers
Beta-NGF	P01138	-0.064	-0.121	-0.011	0.0181	0.4125	Homo-LAV-BPIFB4 carriers
NOTCH1	P46531	-0.060	-0.108	-0.010	0.0187	0.4125	Homo-LAV-BPIFB4 carriers
IL-13	P35225	-0.054	-0.102	-0.008	0.0225	0.4125	Homo-LAV-BPIFB4 carriers
CXCL1	P09341	-0.281	-0.522	-0.040	0.0227	0.4125	Homo-LAV-BPIFB4 carriers
IL-2RB	P14784	-0.065	-0.120	-0.009	0.0236	0.4125	Homo-LAV-BPIFB4 carriers
IFN-gamma	P01579	-0.049	-0.092	-0.007	0.0240	0.4125	Homo-LAV-BPIFB4 carriers
CXCL5	P42830	-0.359	-0.675	-0.047	0.0261	0.4125	Homo-LAV-BPIFB4 carriers
TSLP	Q969D9	-0.105	-0.198	-0.012	0.0264	0.4125	Homo-LAV-BPIFB4 carriers
VCAM1	P19320	-0.077	-0.142	-0.009	0.0266	0.4125	Homo-LAV-BPIFB4 carriers
IL-10RB	Q08334	-0.076	-0.141	-0.009	0.0268	0.4125	Homo-LAV-BPIFB4 carriers
SOD1	P00441	-0.106	-0.198	-0.011	0.0295	0.4352	Homo-LAV-BPIFB4 carriers
LYVE1	Q9Y5Y7	-0.077	-0.149	-0.007	0.0331	0.4594	Homo-LAV-BPIFB4 carriers
IL-6RA	P08887	-0.097	-0.186	-0.008	0.0345	0.4594	Homo-LAV-BPIFB4 carriers
TNFRSF9	Q07011	-0.089	-0.180	-0.006	0.0352	0.4594	Homo-LAV-BPIFB4 carriers
CASP-8	Q14790	-0.218	-0.422	-0.013	0.0363	0.4594	Homo-LAV-BPIFB4 carriers
FCGR3B	O75015	-0.120	-0.233	-0.005	0.0399	0.4867	Homo-LAV-BPIFB4 carriers
REG1A	P05451	-0.111	-0.216	-0.004	0.0424	0.4974	Homo-LAV-BPIFB4 carriers
HGF	P14210	-0.107	-0.206	-0.003	0.0436	0.4974	Homo-LAV-BPIFB4 carriers
TLT-2	Q5T2D2	-0.092	-0.186	0.000	0.0492	0.5299	Homo-LAV-BPIFB4 carriers

Protein symbol = protein symbol; UniprotID = Uniprot protein identifier; Estimate = median of the difference between homozygous LAV-BPIFB4 carriers and others; L95 = lower bound of the 95% Confidence interval (95% CI) of the estimate; U95 = upper bound of the 95% CI of the estimate; BH unadjusted p-value = p-value from the non-parametric Wilcoxon rank sum test not adjusted by the Benjamini-Hochberg correction; BH adjusted p-value = Benjamini-Hochberg adjusted p-value

Gene ontology analyses have been thus performed including, thirty-two genes corresponding to the proteins reaching BH unadjusted p-value < 0.05 from differential secreted analyses (set of genes of interest) and the whole set of 353 proteins with corresponding Entrez gene ID as background genes list.

**Fig. 4:**
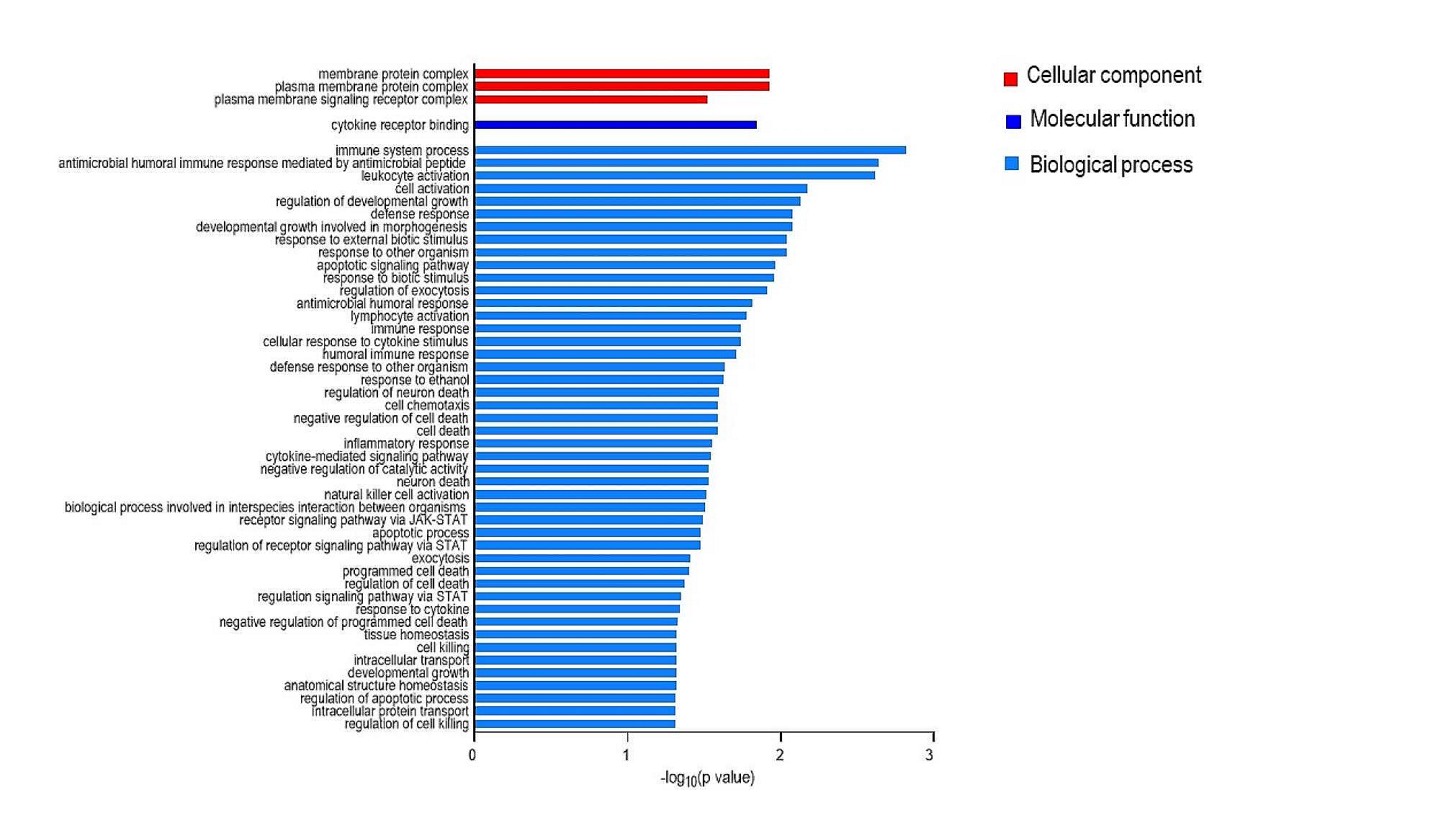
GO terms for BP, MF and CC emerged by differentially circulating protein expression. -log10 unadjusted p-value, proteins showing higher and lower values in homozygous LAV-BPIFB4 carriers compared to others, reaching unadjusted p-value < 0.05, are represented in red and blue, respectively

None of the GO aspects explored (Biological Process, BP; Molecular Function, MF and Cellular Component, CC) showed evidence of statistically significant enrichment after BH adjustment (BH adjusted p-value ≥ 0.05). However, the BP ontology analysis highlighted the “immune system process” as the term showing the strongest evidence of enrichment (BH unadjusted p-value = 0.0015, Fig. 4 and Supplementary Table 3). Moreover, GO underlined a potential overrepresentation of terms associated to infective processes such as “antimicrobial humoral immune response mediated by antimicrobial peptide”, “antimicrobial humoral response”, “defense response to other organism” and “response to external biotic stimulus” (Fig. 4 and Supplementary Table 3). Additionally, there was a possible enrichment of terms associated with CV and cerebrovascular diseases, including “cell chemotaxis” and “activation of leukocyte, lymphocyte and natural killer” and “cell activation” (Fig. 4 and Supplementary Table 3). An overview of the terms further pinpointed the enrichment of cytokine-mediated signalling processes, including JAK-STAT (Fig. 4 and Supplementary Table 3).

As for the MF, the selected extracellular proteins were mainly involved in the cytokine receptor binding (Fig. 4, Supplementary Table 4). The CC included “membrane protein complex”, “plasma membrane protein complex” and “plasma membrane signalling receptor complex” terms (Fig. 4, Supplementary Table 5).

Taken together, the observational analysis underlined an inverse association between the homozygous LAV-BPIFB4 genotype and the immune-inflammatory markers.

### In vitro LAV-BPIFB4 transfer reduces the inflammatory status of IBD

**Fig. 5:**
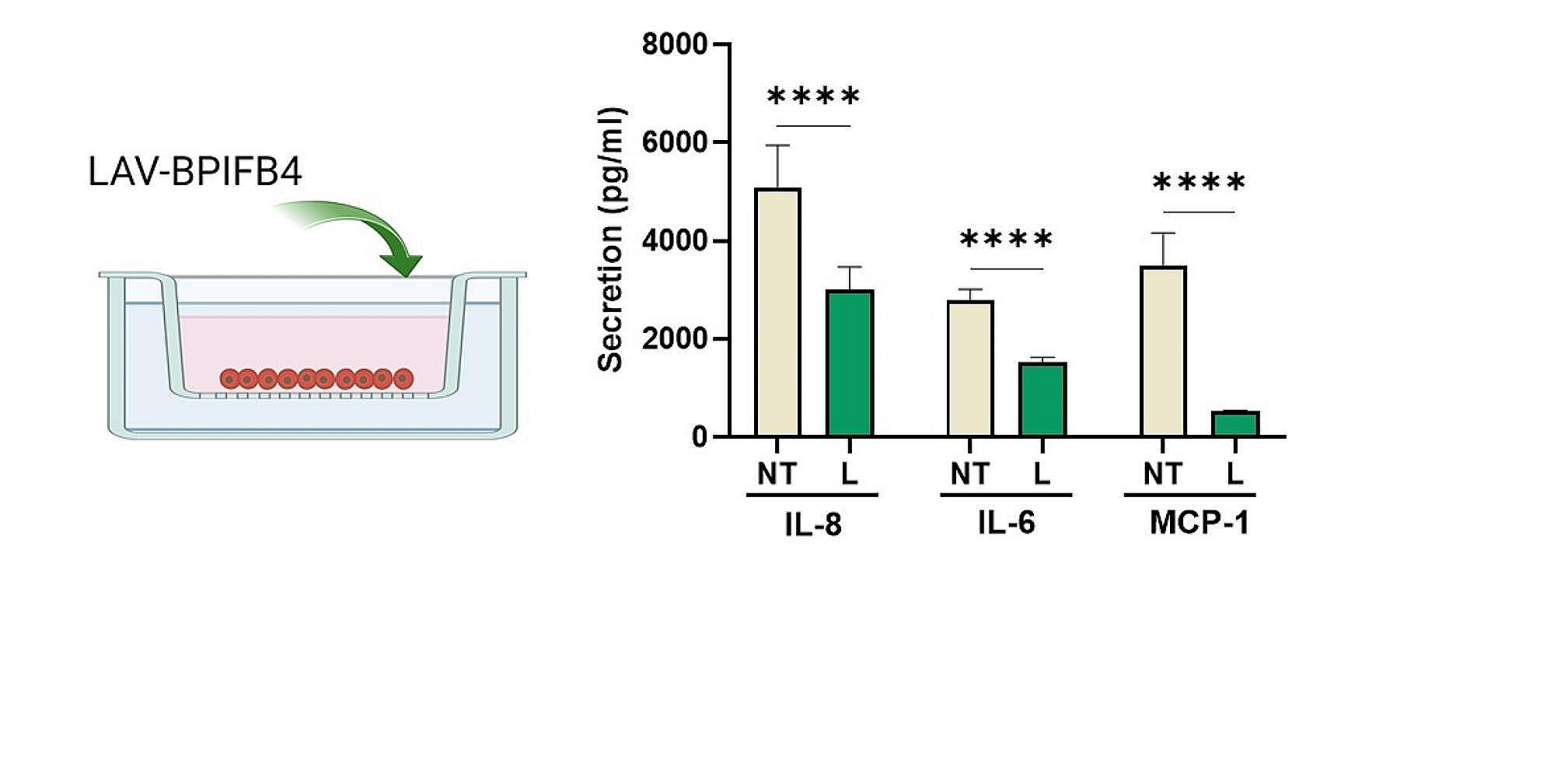
Anti-inflammatory effects of LAV-BPIFB4 exerted on the in vitro intestinal biopsies from IBD patients. Tissue samples from IBD patients were collected. LPS-primed fragments were placed in culture and challenged in absence (NT) or presence of recombinant LAV-BPIFB4 protein (L) (18ng/ml). After 3 h the conditioned medium was collected to assay multiple analytes by beads-based multiplex ELISA. Data were analyzed using Student’s *t*-test (*n* = 6)

Considering the anti-inflammatory effects of homozygous LAV-BPIFB4 genotype and the inflammatory burden characterizing the intestinal epithelial organs derived from IBD patients, we addressed the capacity of the recombinant LAV-BPIFB4 protein to rescue this defect. For this purpose, medium from LPS-primed IBD biopsies cultured in vitro in the presence and absence of recombinant LAV-BPIFB4 was assayed for circulating cytokines levels by beads-based multiplex ELISA. Interestingly, as illustrated in Fig. 5, LAV-BPIFB4 supplementation reduced the levels of IL-6, IL-8 and MCP-1 compared with the control, suggesting a crucial role of the protein in counteracting the immune dysfunction in IBD.

### LAV-BPIFB4 is a responsive protein of the butyrate and enhancer of the histone deacetylase inhibitory activity

Butyrate is considered a therapeutic target in the treatment of IBD due to the capacity to modulate the immune and intestinal barrier functions [22].

**Fig. 6:**
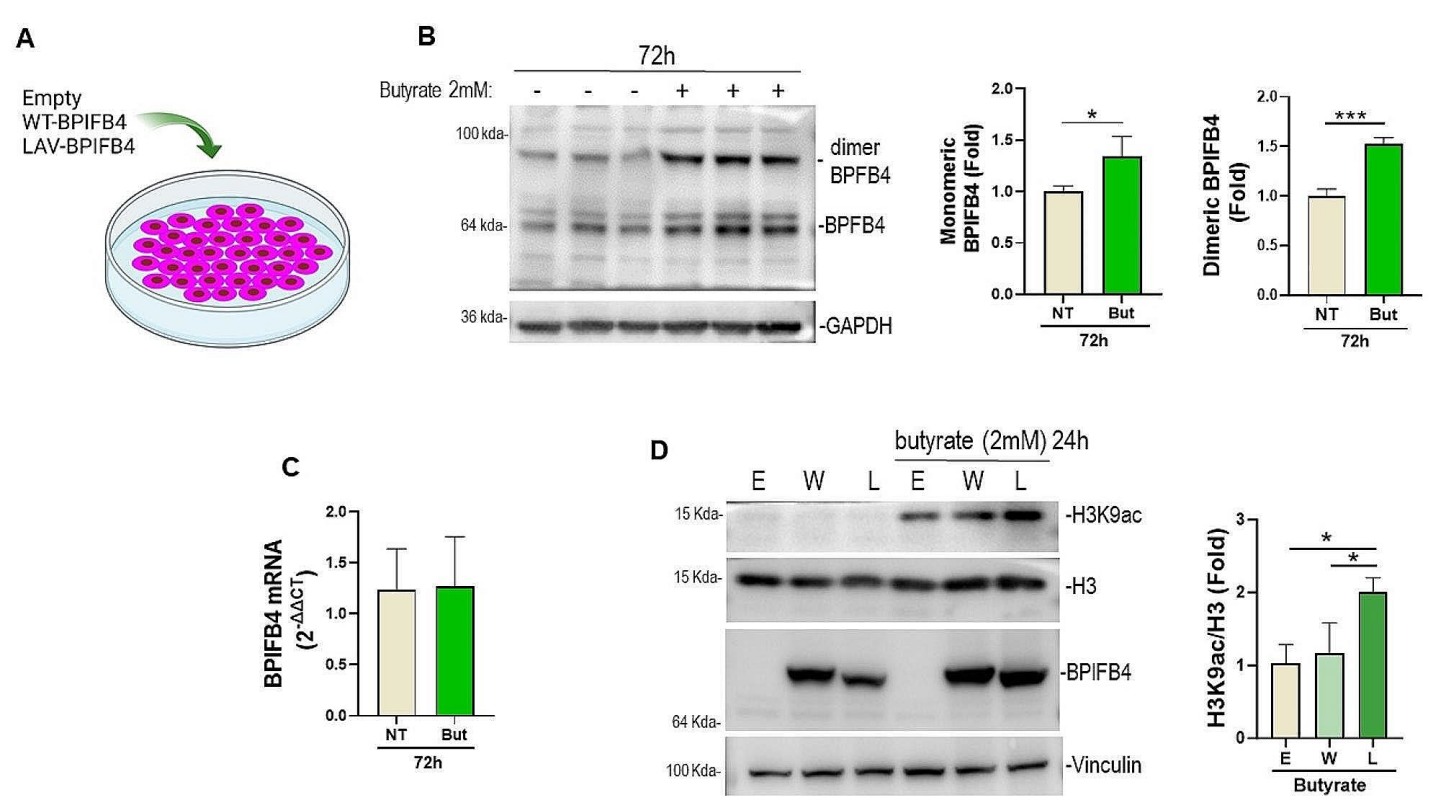
Synergism between LAV-BPIFB4 and butyrate in Caco-2 cells. **A**) Schematic of transfection of BPIFB4 in Caco-2. Endogenous BPIFB4 protein (**B**) and mRNA (**C**) levels in Caco-2 exposed to butyrate treatment (2 mM for 24 h). Data were analyzed using Student’s *t*-test (*n* = 3). **D**) Effect of ectopic BPIFB4 isoforms (W for WT-BPFB4 and L for LAV-BPIFB4) on the acetylation of lysine 9 in H3. Proteins were loaded in non reducing conditions. Empty vector transfected cells are indicated with E. Data were analyzed using two-way ANOVA (*n* = 3)

Thus, we first explored whether therapeutic doses of butyrate could upregulate the endogenous BPIFB4 protein in the human colorectal epithelial adenocarcinoma cell line Caco-2. As shown in Fig. 6A-B, Caco-2 exposed to low dose of butyrate for three days exhibited increased levels of the endogenous monomeric and dimeric BPIFB4 forms. No effect was observed after one day of butyrate treatment (data not shown). Moreover, butyrate did not affect the BPIFB4 mRNA levels (Fig. 6C).

Secondly, we challenged the capacity of ectopic LAV-BPIFB4 protein to potentiate the histone deacetylase inhibitory activity of butyrate. Figure 6D depicts the ability of LAV-BPIFB4 to enhance the effect of butyrate on the acetylation of H3K9 while WT-BPIFB4 was ineffective. To note, transfection reagent and empty vector had no effect on the baseline expression level of H3K9ac compared to the untrasfected cells (Supplementary Fig. 3).

Taken together, the data indicate a feedback loop between LAV-BPIFB4 and butyrate.

## Discussion

Although inflammation plays a critical role in the genesis, progression, and manifestation of CV diseases, the quest for safe and effective therapies targeting inflammation to reduce CV disorders is still ongoing. A better understanding of the genetic drivers negatively impacting inflammation will be of outmost importance to increase our interventional capacity to treat age-related CV disease, eventually improving the quality and extension of life. In this contest, the homozygous LAV-BPIFB4 carriers represent a precious source of biological information to be carefully studied. Indeed, being associated with healthy cardiovascular ageing and immunomodulatory properties, LAV-BPIFB4 may represent valuable therapeutic tools to counteract the decline of CV function related to inflammation. Thus, studying carriers of homozygous LAV-BPIFB4 genotype could shed light on the immune-phenotype that should contribute to the resistance of aged-related CV diseases.

**Fig. 7:**
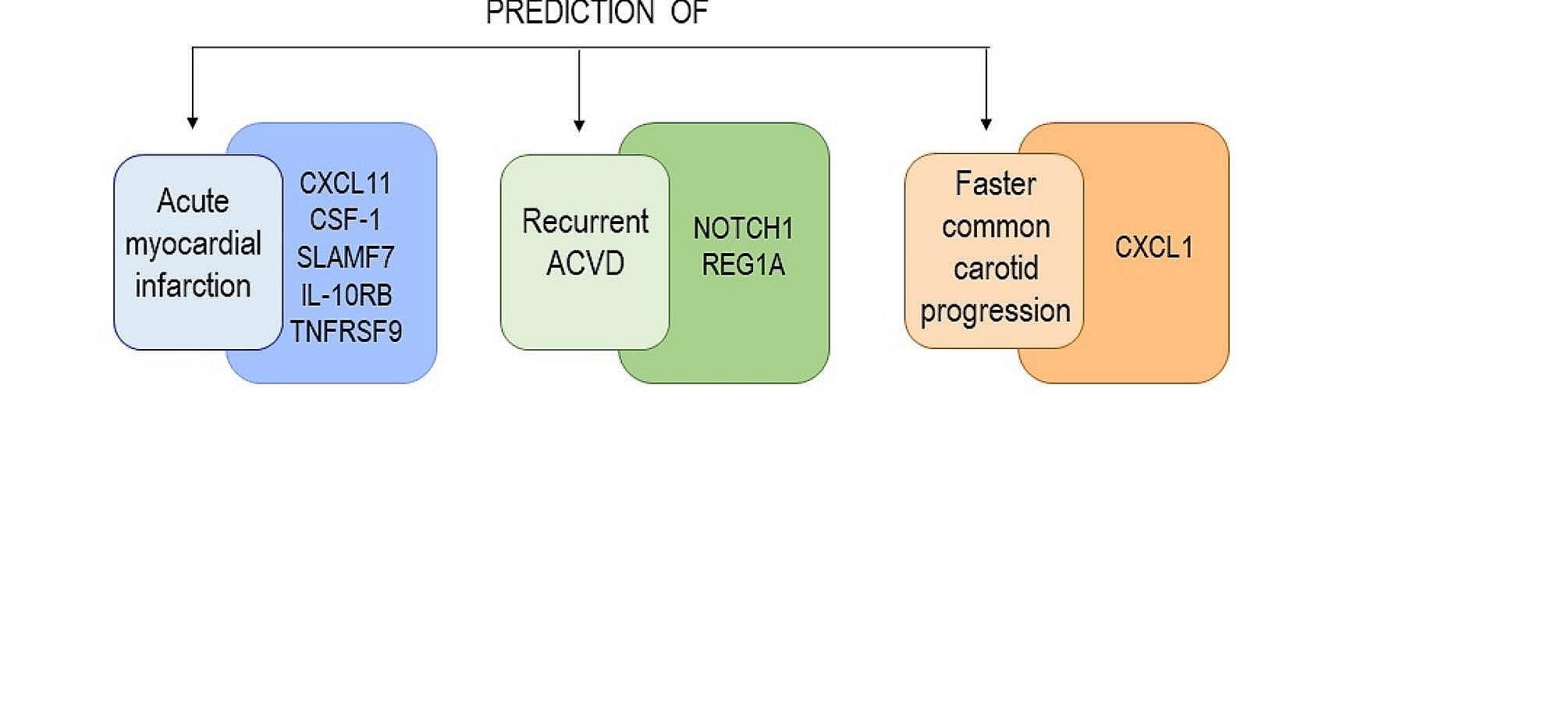
Proteins with predictive values for CV diseases. Schematic representation of downmodulated proteins in LAV-BPIFB4 that have been emerged as predictive factors for CV diseases according to previous proteome-based CV risk models

By the use of our proteomics analysis, we identified thirty-one proteins under-expressed in homozygous LAV-BPIFB4 carriers compared to the other BPIFB4 genotypes, strengthening that inheriting the longevity variant may result into reduced inflammatory burden lifelong. Five proteins deregulated in homozygous LAV-BPIFB4 carriers (e.g. CXCL11, CSF-1, SLAMF7, IL-10RB and TNFRSF9) emerged from a recent proteome-based CV risk model as the top factors predicting myocardial infarction event [18] (Fig. 7). Furthermore, NOTCH1 and REG1A were found among the proteins with the strongest predictive value for atherosclerotic cardiovascular events [23] while CXCL1 as the top predictive protein for the faster common carotid progression [16] (Fig. 7). These findings bolster the concept that the homozygous LAV-BPIFB4 genotype may confer protection against aged-related CV diseases, making it a shining example of positive biology.

Concerning ITGAM, the exclusive upregulated protein in LAV-BPIFB4 genotype with anti-inflammatory property, is one of the main SLE-susceptibility loci. Several studies have shown that the non-synonymous *ITGAM* rs1143679G/A SNP affects the structure and function of the protein and also decreases the expression levels of the transcript and the protein [20]. In this contest, the ITGAM downmodulation observed in the subjects not carried the homozygous LAV-BPIFB4 genotype could account for a defective immune-circulating function.

Interestingly, LAV-BPIFB4 caused a remarkable change in the paracrine repertoire of colonic biopsies derived from IBD patients, reducing the release of inflammatory cytokines. This paracrine response together with the proteome profile of PLIC cohort reinforced the evidence on the immunomodulatory proprieties exhibited by LAV-BPIFB4. Consistently, Fujimori et al. reported that five SNPs in BPIFB4, including rs2070325, are associated with non-steroidal anti-inflammatory drugs (NSAIDs)-induced small intestinal mucosal injury supporting promising evidence that LAV-BPIFB4 haplotype may be useful for the defence against small intestinal ulcerative lesions caused by NSAIDs [24].We newly show that endogenous BPIFB4 protein was positive regulated by butyrate, a short chain fatty acid able, at appropriate concentrations, to maintain intestinal barrier function and regulate the immune response by distinct transcriptional regulatory mechanisms, including inhibition of NF-κB and histone deacetylase activity [3, 22, 25]. Although the levels of BPIFB4 protein increased, the amount of the transcript was unaltered, indicating a post-translational protein stabilization induced by butyrate treatment, a mechanism previously described [26]. Interestingly, ectopic LAV-BPIFB4 in Caco-2 potentiated the histone deacetylase inhibitory activity of butyrate. This information complements our previous reports regarding the involvement of LAV-BPIFB4 in the chromatin remodeling [10, 11].

Several limitations to our study merit discussion. First, the number of the subjects from PLIC cohort enrolled in the analysis. The lack of statistically significant differences in the protein expression between the two genotypic groups when adjusted by BH correction could be due to the limited number of analysed patients (*n* = 591) and by the presence of potential confounding factors. Multivariate quantile regression showed that the difference in terms of TNFSF14 protein concentration between genotype groups was not influenced by sex and LDL-C values, identified as measured potential confounders. However, since patients have been not selected to be included in the analysed cohort based on their genotype information, we can hypothesize that population stratification should not represent a major bias in the present study. A larger cohort characterized by sufficient statistical power to detect the expected effect sizes using a multiple testing corrected significance level and the inclusion of clinical and demographic data in suitable multivariate regression models could confirm the current results and help identifying informative interactions between genetic and non-genetic information potentially modulating proteins expression.

Second, the use of panels specialized for proteins associated with biological functions linked to CV diseases and inflammation might drastically influence the GO analyses with the result to obtain an overrepresentation of categories associated to preselected diseases. Despite this, the intent of our GO analyses was to provide an explorative list of the key representative pathways within the pre-decided disorders. An example is given by JAK-STAT axis which emerged as unique enriched molecular pathway within the cytokine-mediated signalling. Finally, by using targeted than untargeted proteomics, proteins not included in the panels may have been missed with the result to obtain an underestimation of LAV-BPIFB4 signature.

Concerning the technical performance of Olink PEA technology, several publications report the high-quality data obtained by this innovative technique and the great impact in the field of protein biomarkers. Notably, Olink PEA technology provides exceptional read-out specificity. The use of dual antibodies with coupled DNA-tags that have to be in close proximity, eliminates cross-reactive events, a common problem for multiplex immunoassays, providing exceptional specificity even at high multiplexing levels [27]. Moreover, Olink’s multiplex assays correlate well with single-plex fully automated ELISA used in clinical laboratories for routine analysis. Indeed, Siegbahn et al. reported a strong correlation between Olink 92-plex PEA assay compared to single-plex ELISA for IL-6, CXCL-10 and GDF-15 [28]. In accordance, the pattern profile of the top dowmnodulated factor TNFSF14 identified by Olink technology, has been corroborated by ELISA, strengthening the technical robustness and reliable performance of this technology and its utility for simultaneous evaluation of many blood-based candidate biomarkers.

**Fig. 8:**
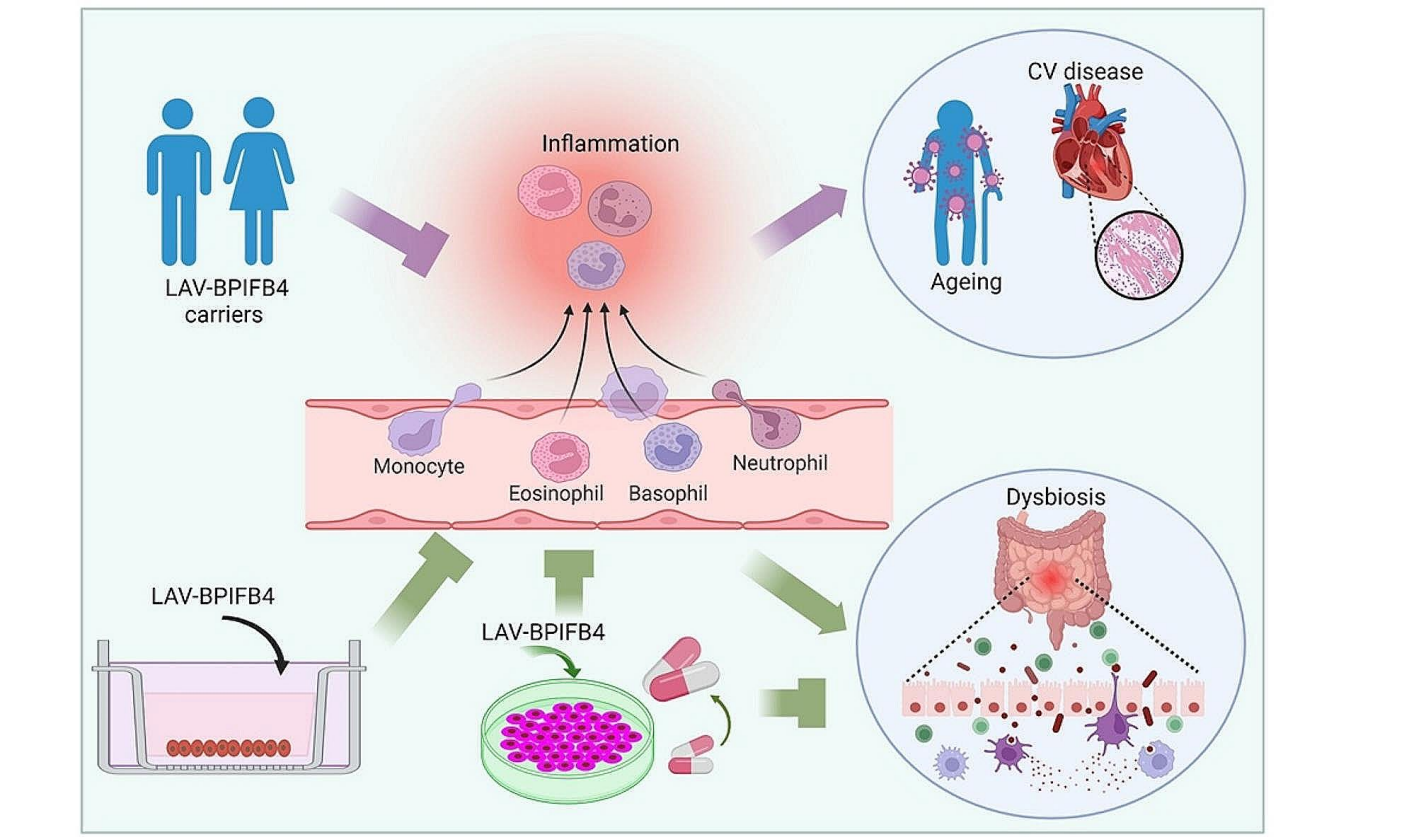
**Schematic summarizes inflammation process occurring in multiple diseases that can be attenuated by LAV-BPIFB4.** Ageing, CV disease and IBD are characterized by chronic low-grade inflammation. Homozygosity for LAV-BPIFB4 reduced expression of plasmatic markers related to inflammation

In the present study, we showed an inverse association between LAV-BPIFB4 homozygous genotype and markers related to inflammation, a remarkably benefit of LAV-BPIFB4 supplementation on colonic biopsies derived from IBD patients and a LAV-BPIFB4 favourable effect on the butyrate activity (Fig. 8).

## Conclusion

Both genetic variation and environmental factors may influence pro-inflammatory response and eventual resilience or susceptibility to chronic inflammatory diseases. This study provides evidence for a protective role of LAV-BPIFB4 genotype in relation to ageing inflammation supporting the notion that transferring the healthy trait to individuals with inflammatory diseases may help their immune system to maintain correct function. However, results presented in this study must be considered as preliminary and hypothesis generating, being likely affected by false positive findings given the influence of potentially unmeasured confounding factors and the limited number of the subjects investigated. The results described in this study need therefore to be confirmed by the analysis of independent and adequately powered cohorts before confirming their relevance.

### Electronic supplementary material

Below is the link to the electronic supplementary material.

Supplementary Material 1

Supplementary Material 2

Supplementary Material 3

Supplementary Material 4

Supplementary Material 5

Supplementary Material 6

Supplementary Material 7

Supplementary Material 8

## Data Availability

No datasets were generated or analysed during the current study.

## References

[1] Vasto S, Candore G, Balistreri CR (2007). Inflammatory networks in ageing, age-related diseases and longevity. Mech Ageing Dev.

[2] Bonacina F, Baragetti A, Catapano AL, Norata GD (2019). The interconnection between Immuno-Metabolism, Diabetes, and CKD. Curr Diab Rep.

[3] Recharla N, Geesala R, Shi XZ (2023). Gut Microbial Metabolite Butyrate and its therapeutic role in inflammatory bowel disease: a Literature Review. Nutrients.

[4] Haran JP, McCormick BA, Aging (2021). Frailty, and the Microbiome-How Dysbiosis influences Human Aging and Disease. Gastroenterology.

[5] Dahal RH, Kim S, Kim YK, Kim ES, Kim J (2023). Insight into gut dysbiosis of patients with inflammatory bowel disease and ischemic colitis. Front Microbiol.

[6] Villa F, Carrizzo A, Spinelli CC (2015). Genetic analysis reveals a Longevity-Associated protein modulating endothelial function and angiogenesis. Circ Res.

[7] Vecchione C, Villa F, Carrizzo A et al. Author Correction: A rare genetic variant of BPIFB4 predisposes to high blood pressure via impairment of nitric oxide signaling. Sci Rep. 2019;9(1):9574. Published 2019. 10.1038/s41598-019-45691-1. 10.1038/s41598-019-45691-1 PMC6597558 31249326

[8] Villa F, Carrizzo A, Ferrario A (2018). A model of Evolutionary Selection: the Cardiovascular Protective function of the Longevity Associated variant of BPIFB4. Int J Mol Sci.

[9] Puca AA, Carrizzo A, Spinelli C (2020). Single systemic transfer of a human gene associated with exceptional longevity halts the progression of atherosclerosis and inflammation in ApoE knockout mice through a CXCR4-mediated mechanism. Eur Heart J.

[10] Cattaneo M, Beltrami AP, Thomas AC (2023). The longevity-associated BPIFB4 gene supports cardiac function and vascularization in ageing cardiomyopathy. Cardiovasc Res.

[11] Cattaneo M, Aleksova A, Malovini A (2023). BPIFB4 and its longevity-associated haplotype protect from cardiac ischemia in humans and mice. Cell Death Dis.

[12] Ciaglia E, Lopardo V, Montella F (2022). Transfer of the longevity-associated variant of BPIFB4 gene rejuvenates immune system and vasculature by a reduction of CD38 + macrophages and NAD + decline. Cell Death Dis.

[13] Malavolta M, Dato S, Villa F (2019). Correction for: LAV-BPIFB4 associates with reduced frailty in humans and its transfer prevents frailty progression in old mice. Aging.

[14] Giuliani ME, Barbi V, Bigossi G (2023). Effects of Human LAV-BPIFB4 Gene Therapy on the epigenetic clock and health of aged mice. Int J Mol Sci.

[15] Di Pardo A, Ciaglia E, Cattaneo M (2020). The longevity-associated variant of BPIFB4 improves a CXCR4-mediated striatum-microglia crosstalk preventing disease progression in a mouse model of Huntington’s disease. Cell Death Dis.

[16] Baragetti A, Mattavelli E, Grigore L, Pellegatta F, Magni P, Catapano AL (2022). Targeted plasma proteomics to predict the development of carotid plaques. Stroke.

[17] Olmastroni E, Baragetti A, Casula M (2019). Multilevel models to Estimate Carotid Intima-Media Thickness curves for Individual Cardiovascular Risk evaluation. Stroke.

[18] Hoogeveen RM, Pereira JPB, Nurmohamed NS (2020). Improved cardiovascular risk prediction using targeted plasma proteomics in primary prevention. Eur Heart J.

[19] Khan SQ, Khan I, Gupta V (2018). CD11b activity modulates pathogenesis of Lupus Nephritis. Front Med (Lausanne).

[20] Ramírez-Bello J, Sun C, Valencia-Pacheco G (2019). ITGAM is a risk factor to systemic lupus erythematosus and possibly a protection factor to rheumatoid arthritis in patients from Mexico. PLoS ONE.

[21] Mauri DN, Ebner R, Montgomery RI (1998). LIGHT, a new member of the TNF superfamily, and lymphotoxin alpha are ligands for herpesvirus entry mediator. Immunity.

[22] Hodgkinson K, El Abbar F, Dobranowski P (2023). Butyrate’s role in human health and the current progress towards its clinical application to treat gastrointestinal disease. Clin Nutr.

[23] Nurmohamed NS, Belo Pereira JP, Hoogeveen RM (2022). Targeted proteomics improves cardiovascular risk prediction in secondary prevention. Eur Heart J.

[24] Fujimori S, Fukunaga K, Takahashi A (2019). Bactericidal/Permeability-Increasing fold-containing Family B Member 4 May be Associated with NSAID-Induced Enteropathy. Dig Dis Sci.

[25] Lee C, Kim BG, Kim JH, Chun J, Im JP, Kim JS (2017). Sodium butyrate inhibits the NF-kappa B signaling pathway and histone deacetylation, and attenuates experimental colitis in an IL-10 independent manner. Int Immunopharmacol.

[26] Nakagawa H, Sasagawa S, Itoh K (2018). Sodium butyrate induces senescence and inhibits the invasiveness of glioblastoma cells. Oncol Lett.

[27] Assarsson E, Lundberg M, Holmquist G et al. Homogenous 96-plex PEA immunoassay exhibiting high sensitivity, specificity, and excellent scalability. PLoS One. 2014;9(4):e95192. Published 2014 Apr 22. 10.1371/journal.pone.0095192. 10.1371/journal.pone.0095192 PMC3995906 24755770

[28] Siegbahn A, Eriksson A, Lindbäck J, Wallentin L. A comparison of the proximity extension assay with established immunoassays. Advancing precision medicine: current and future proteogenomic strategies for biomarker discovery and development. Science/AAAS; 2017. pp. 22–5.

