# Supplementary figures and images for: Longevity-associated BPIFB4 gene counteracts the inflammatory signaling
Source: Immun Ageing. 2024 Mar 12;21:19. doi: 10.1186/s12979-024-00424-5 (PMC10929107; doi:10.1186/s12979-024-00424-5)

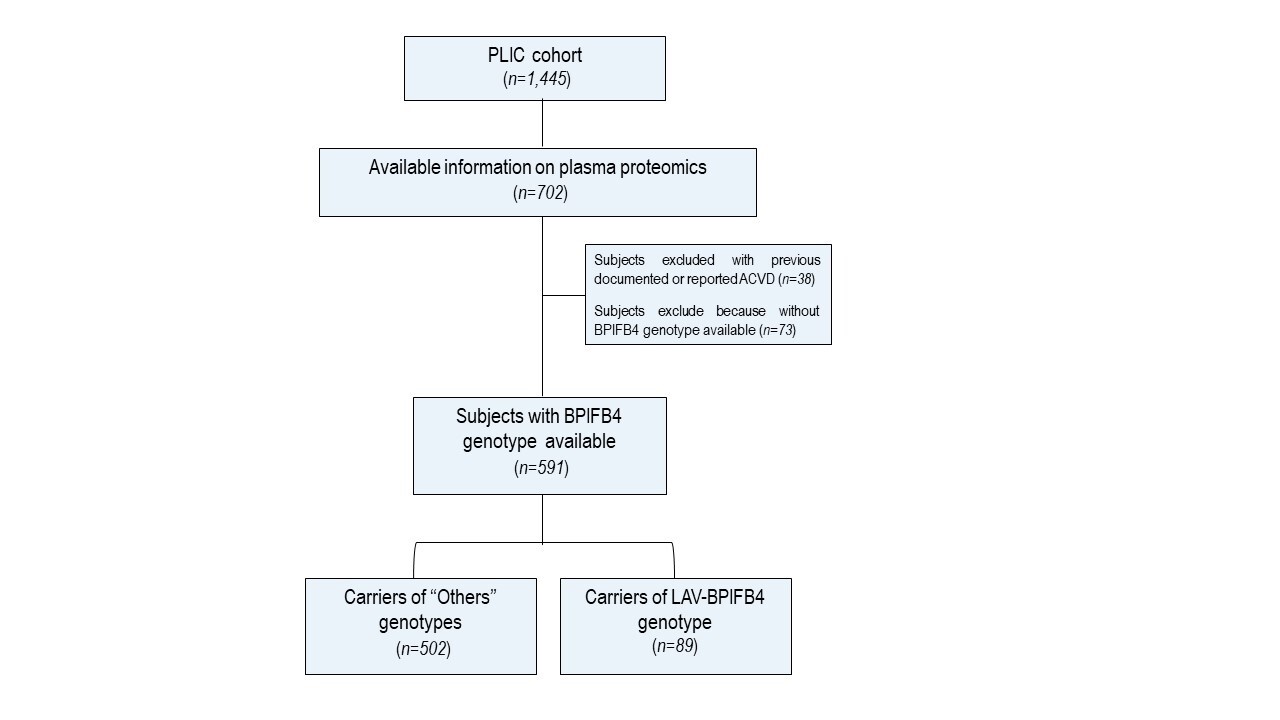

Supplement: Supplementary file 1 — Supplementary Material 1 [file 12979_2024_424_MOESM1_ESM.jpg]

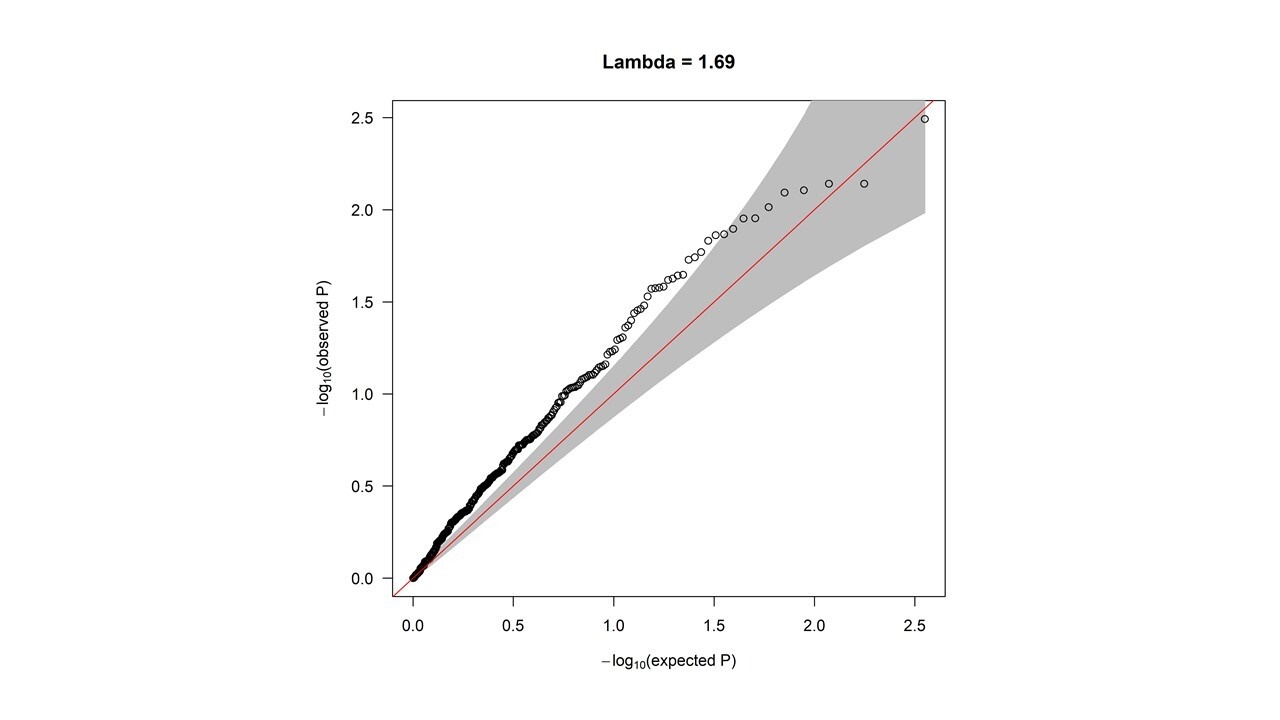

Supplement: Supplementary file 2 — Supplementary Material 2 [file 12979_2024_424_MOESM2_ESM.jpg]

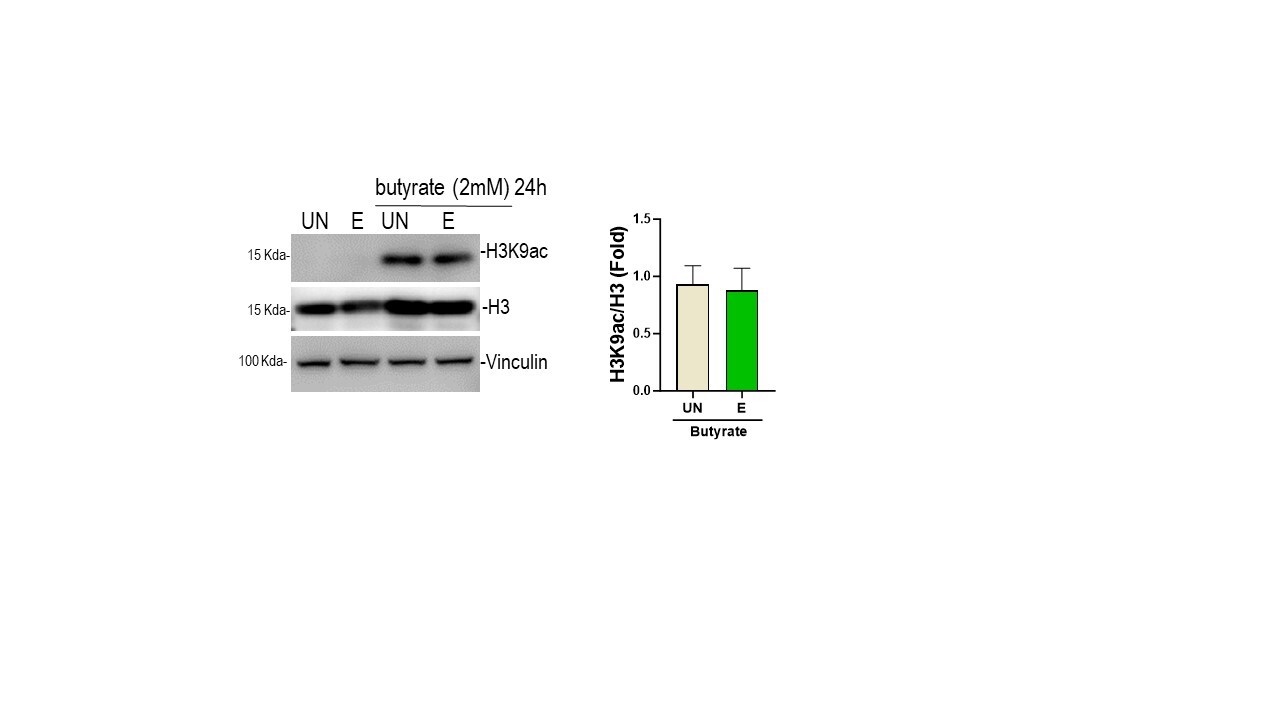

Supplement: Supplementary file 3 — Supplementary Material 3 [file 12979_2024_424_MOESM3_ESM.jpg]
