# Supplementary material for: Longevity-associated BPIFB4 gene counteracts the inflammatory signaling
Source: Immun Ageing. 2024 Mar 12;21:19. doi: 10.1186/s12979-024-00424-5 (PMC10929107; doi:10.1186/s12979-024-00424-5)
Supplement: Supplementary file 4 — Supplementary Material 4 [file 12979_2024_424_MOESM4_ESM.docx]

| **Protein symbol** | **Protein name** | **Uniprot ID** | **Gene symbol** | **Entrez gene ID** | **Estimate** | **L95** | **U95** | **BH unadjusted p-value** | **BH adjusted p-value** |
| --- | --- | --- | --- | --- | --- | --- | --- | --- | --- |
| TNFSF14 | Tumor necrosis factor ligand superfamily member 14 | O43557 | *TNFSF14* | 8740 | -0.206 | -0.339 | -0.070 | 0.0032 | 0.4125 |
| PTPRS | Receptor-type tyrosine-protein phosphatase S | Q13332 | *PTPRS* | 5802 | -0.062 | -0.106 | -0.017 | 0.0072 | 0.4125 |
| CXCL11 | C-X-C motif chemokine 11 | O14625 | *CXCL11* | 6373 | -0.289 | -0.494 | -0.083 | 0.0072 | 0.4125 |
| CXCL6 | C-X-C motif chemokine 6 | P80162 | *CXCL6* | 6372 | -0.273 | -0.481 | -0.072 | 0.0078 | 0.4125 |
| ITGAM | Integrin alpha-M | P11215 | *ITGAM* | 3684 | 0.092 | 0.025 | 0.159 | 0.0081 | 0.4125 |
| CD244 | Natural killer cell receptor 2B4 | Q9BZW8 | *CD244* | 51744 | -0.117 | -0.202 | -0.030 | 0.0097 | 0.4125 |
| IL-20RA | Interleukin-20 receptor subunit alpha | Q9UHF4 | *IL20RA* | 53832 | -0.064 | -0.112 | -0.015 | 0.0111 | 0.4125 |
| PRCP | Lysosomal Pro-X carboxypeptidase | P42785 | *PRCP* | 5547 | -0.093 | -0.166 | -0.022 | 0.0112 | 0.4125 |
| REG3A | Regenerating islet-derived protein 3-alpha | Q06141 | *REG3A* | 5068 | -0.062 | -0.110 | -0.013 | 0.0127 | 0.4125 |
| HSP 27 | Heat shock 27 kDa protein | P04792 | *HSPB1* | 3315 | -0.131 | -0.229 | -0.027 | 0.0136 | 0.4125 |
| CSF-1 | Macrophage colony-stimulating factor 1 | P09603 | *CSF1* | 1435 | -0.077 | -0.135 | -0.016 | 0.0137 | 0.4125 |
| SLAMF7 | SLAM family member 7 | Q9NQ25 | *SLAMF7* | 57823 | -0.116 | -0.209 | -0.022 | 0.0147 | 0.4125 |
| IDUA | Alpha-L-iduronidase | P35475 | *IDUA* | 3425 | -0.252 | -0.455 | -0.046 | 0.0170 | 0.4125 |
| Beta-NGF | Beta-nerve growth factor | P01138 | *NGF* | 4803 | -0.064 | -0.121 | -0.011 | 0.0181 | 0.4125 |
| NOTCH1 | Neurogenic locus notch homolog protein 1 | P46531 | *NOTCH1* | 4851 | -0.060 | -0.108 | -0.010 | 0.0187 | 0.4125 |
| IL-13 | Interleukin-13 | P35225 | *IL13* | 3596 | -0.054 | -0.102 | -0.008 | 0.0225 | 0.4125 |
| CXCL1 | C-X-C motif chemokine 1 | P09341 | *CXCL1* | 2919 | -0.281 | -0.522 | -0.040 | 0.0227 | 0.4125 |
| IL-2RB | Interleukin-2 receptor subunit beta | P14784 | *IL2RB* | 3560 | -0.065 | -0.120 | -0.009 | 0.0236 | 0.4125 |
| IFN-gamma | Interferon gamma | P01579 | *IFNG* | 3458 | -0.049 | -0.092 | -0.007 | 0.0240 | 0.4125 |
| CXCL5 | C-X-C motif chemokine 5 | P42830 | *CXCL5* | 6374 | -0.359 | -0.675 | -0.047 | 0.0261 | 0.4125 |
| TSLP | Thymic stromal lymphopoietin | Q969D9 | *TSLP* | 85480 | -0.105 | -0.198 | -0.012 | 0.0264 | 0.4125 |
| VCAM1 | Vascular cell adhesion protein 1 | P19320 | *VCAM1* | 7412 | -0.077 | -0.142 | -0.009 | 0.0266 | 0.4125 |
| IL-10RB | Interleukin-10 receptor subunit beta | Q08334 | *IL10RB* | 3588 | -0.076 | -0.141 | -0.009 | 0.0268 | 0.4125 |
| SOD1 | Superoxide dismutase [Cu-Zn] | P00441 | *SOD1* | 6647 | -0.106 | -0.198 | -0.011 | 0.0295 | 0.4352 |
| LYVE1 | Lymphatic vessel endothelial hyaluronic acid receptor 1 | Q9Y5Y7 | *LYVE1* | 10894 | -0.077 | -0.149 | -0.007 | 0.0331 | 0.4594 |
| IL-6RA | Interleukin-6 receptor subunit alpha | P08887 | *IL6R* | 3570 | -0.097 | -0.186 | -0.008 | 0.0345 | 0.4594 |
| TNFRSF9 | Tumor necrosis factor receptor superfamily member 9 | Q07011 | *TNFRSF9* | 3604 | -0.089 | -0.180 | -0.006 | 0.0352 | 0.4594 |
| CASP-8 | Caspase-8 | Q14790 | *CASP8* | 841 | -0.218 | -0.422 | -0.013 | 0.0363 | 0.4594 |
| FCGR3B | Low affinity immunoglobulin gamma Fc region receptor III-B | O75015 | *FCGR3B* | 2215 | -0.120 | -0.233 | -0.005 | 0.0399 | 0.4867 |
| REG1A | Lithostathine-1-alpha | P05451 | *REG1A* | 5967 | -0.111 | -0.216 | -0.004 | 0.0424 | 0.4974 |
| HGF | Hepatocyte growth factor | P14210 | *HGF* | 3082 | -0.107 | -0.206 | -0.003 | 0.0436 | 0.4974 |
| TLT-2 | Trem-like transcript 2 protein | Q5T2D2 | *TREML2* | 79865 | -0.092 | -0.186 | 0.000 | 0.0492 | 0.5299 |
| GDF-2 | Growth/differentiation factor 2 | Q9UK05 | *GDF2* | 2658 | -0.127 | -0.248 | 0.000 | 0.0501 | 0.5299 |
| LIF-R | Leukemia inhibitory factor receptor | P42702 | *LIFR* | 3977 | -0.052 | -0.102 | 0.000 | 0.0509 | 0.5299 |
| SERPINA7 | Thyroxine-binding globulin | P05543 | *SERPINA7* | 6906 | -0.080 | -0.160 | 0.002 | 0.0573 | 0.5612 |
| CCL18 | C-C motif chemokine 18 | P55774 | *CCL18* | 6362 | 0.146 | -0.005 | 0.300 | 0.0586 | 0.5612 |
| Dkk-1 | Dickkopf-related protein 1 | O94907 | *DKK1* | 22943 | -0.167 | -0.347 | 0.007 | 0.0590 | 0.5612 |
| SELL | L-selectin | P14151 | *SELL* | 6402 | -0.068 | -0.138 | 0.003 | 0.0611 | 0.5612 |
| SIRT2 | SIR2-like protein 2 | Q8IXJ6 | *SIRT2* | 22933 | -0.231 | -0.481 | 0.019 | 0.0690 | 0.5612 |
| AGRP | Agouti-related protein | O00253 | *AGRP* | 181 | 0.107 | -0.009 | 0.226 | 0.0706 | 0.5612 |
| DEFA1 | Neutrophil defensin 1 | P59665 | *DEFA1* | 1667 | -0.029 | -0.061 | 0.003 | 0.0710 | 0.5612 |
| TGF-alpha | Transforming growth factor alpha | P01135 | *TGFA* | 7039 | -0.119 | -0.251 | 0.010 | 0.0719 | 0.5612 |
| CXCL9 | C-X-C motif chemokine 9 | Q07325 | *CXCL9* | 4283 | -0.128 | -0.269 | 0.013 | 0.0743 | 0.5612 |
| PTX3 | Pentraxin-related protein PTX3 | P26022 | *PTX3* | 5806 | -0.084 | -0.175 | 0.009 | 0.0768 | 0.5612 |
| TNFRSF10C | Tumor necrosis factor receptor superfamily member 10C | O14798 | *TNFRSF10C* | 8794 | -0.104 | -0.218 | 0.011 | 0.0787 | 0.5612 |
| PD-L1 | Programmed cell death 1 ligand 1 | Q9NZQ7 | *CD274* | 29126 | -0.069 | -0.148 | 0.008 | 0.0788 | 0.5612 |
| STAMBP | STAM-binding protein | O95630 | *STAMBP* | 10617 | -0.177 | -0.374 | 0.021 | 0.0789 | 0.5612 |
| TFPI | Tissue factor pathway inhibitor | P10646 | *TFPI* | 7035 | 0.064 | -0.007 | 0.136 | 0.0808 | 0.5612 |
| LCN2 | Neutrophil gelatinase-associated lipocalin | P80188 | *LCN2* | 3934 | -0.059 | -0.128 | 0.008 | 0.0817 | 0.5612 |
| IL-8 | Interleukin-8 | P10145 | *CXCL8* | 3576 | -0.118 | -0.253 | 0.015 | 0.0827 | 0.5612 |
| HB-EGF | Proheparin-binding EGF-like growth factor | Q99075 | *HBEGF* | 1839 | -0.191 | -0.406 | 0.026 | 0.0835 | 0.5612 |
| TNFRSF13B | Tumor necrosis factor receptor superfamily member 13B | O14836 | *TNFRSF13B* | 23495 | -0.070 | -0.154 | 0.010 | 0.0864 | 0.5612 |
| KLK6 | Kallikrein-6 | Q92876 | *KLK6* | 5653 | 0.067 | -0.010 | 0.143 | 0.0896 | 0.5612 |
| CX3CL1 | Fractalkine | P78423 | *CX3CL1* | 6376 | -0.081 | -0.175 | 0.013 | 0.0902 | 0.5612 |
| CEACAM8 | Carcinoembryonic antigenrelated cell adhesion molecule 8 | P31997 | *CEACAM8* | 1088 | -0.115 | -0.245 | 0.017 | 0.0919 | 0.5612 |
| IL-1ra | Interleukin-1 receptor antagonist protein | P18510 | *IL1RN* | 3557 | -0.143 | -0.313 | 0.025 | 0.0921 | 0.5612 |
| FCN2 | Ficolin-2 | Q15485 | *FCN2* | 2220 | 0.112 | -0.017 | 0.245 | 0.0924 | 0.5612 |
| IL-7 | Interleukin-7 | P13232 | *IL7* | 3574 | -0.148 | -0.323 | 0.026 | 0.0929 | 0.5612 |
| GT | Gastrotropin | P51161 | *FABP6* | 2172 | 0.119 | -0.021 | 0.265 | 0.0940 | 0.5612 |
| LEP | Leptin | P41159 | *LEP* | 3952 | -0.212 | -0.461 | 0.038 | 0.0954 | 0.5612 |
| LOX-1 | Lectin-like oxidized LDL receptor 1 | P78380 | *OLR1* | 4973 | -0.131 | -0.283 | 0.023 | 0.0967 | 0.5612 |
| PDGF subunit B | Platelet-derived growth factor subunit B | P01127 | *PDGFB* | 5155 | -0.156 | -0.342 | 0.032 | 0.1017 | 0.5691 |
| GNLY | Granulysin | P22749 | *GNLY* | 10578 | -0.053 | -0.115 | 0.010 | 0.1027 | 0.5691 |
| TIE1 | Tyrosine-protein kinase receptor Tie-1 | P35590 | *TIE1* | 7075 | -0.040 | -0.088 | 0.008 | 0.1029 | 0.5691 |
| LTBP2 | Latent-transforming growth factor beta-binding protein 2 | Q14767 | *LTBP2* | 4053 | -0.048 | -0.107 | 0.011 | 0.1109 | 0.5909 |
| MCP-4 | Monocyte chemotactic protein 4 | Q99616 | *CCL13* | 6357 | -0.124 | -0.273 | 0.028 | 0.1109 | 0.5909 |
| MMP-3 | Matrix metalloproteinase-3 | P08254 | *MMP3* | 4314 | 0.132 | -0.031 | 0.297 | 0.1118 | 0.5909 |
| IL-2 | Interleukin-2 | P60568 | *IL2* | 3558 | -0.047 | -0.105 | 0.011 | 0.1171 | 0.6096 |
| TF | Tissue factor | P13726 | *F3* | 2152 | -0.057 | -0.130 | 0.016 | 0.1193 | 0.6122 |
| PRTN3 | Myeloblastin | P24158 | *PRTN3* | 5657 | -0.087 | -0.197 | 0.022 | 0.1224 | 0.6189 |
| NEMO | NF-kappa-B essential modulator | Q9Y6K9 | *IKBKG* | 8517 | -0.141 | -0.333 | 0.041 | 0.1258 | 0.6275 |
| IL-27 | Interleukin-27 | Q8NEV9 | *IL27* | 246778 | -0.084 | -0.194 | 0.026 | 0.1302 | 0.6370 |
| QPCT | Glutaminyl-peptide cyclotransferase | Q16769 | *QPCT* | 25797 | -0.049 | -0.112 | 0.015 | 0.1314 | 0.6370 |
| IL-4RA | Interleukin-4 receptor subunit alpha | P24394 | *IL4R* | 3566 | -0.045 | -0.104 | 0.014 | 0.1344 | 0.6385 |
| CTRC | Chymotrypsin C | Q99895 | *CTRC* | 11330 | -0.112 | -0.257 | 0.034 | 0.1353 | 0.6385 |
| MCP-2 | Monocyte chemotactic protein 2 | P80075 | *CCL8* | 6355 | -0.108 | -0.254 | 0.037 | 0.1390 | 0.6400 |
| REN | Renin | P00797 | *REN* | 5972 | 0.128 | -0.041 | 0.298 | 0.1406 | 0.6400 |
| SRC | Proto-oncogene tyrosine-protein kinase Src | P12931 | *SRC* | 6714 | -0.156 | -0.373 | 0.056 | 0.1425 | 0.6400 |
| HAOX1 | Hydroxyacid oxidase 1 | Q9UJM8 | *HAO1* | 54363 | 0.190 | -0.061 | 0.438 | 0.1452 | 0.6400 |
| FETUB | Fetuin-B | Q9UGM5 | *FETUB* | 26998 | -0.058 | -0.138 | 0.021 | 0.1469 | 0.6400 |
| SORT1 | Sortilin | Q99523 | *SORT1* | 6272 | -0.062 | -0.142 | 0.021 | 0.1476 | 0.6400 |
| CHL1 | Neural cell adhesion molecule L1-like protein | O00533 | *CHL1* | 10752 | -0.054 | -0.129 | 0.021 | 0.1536 | 0.6400 |
| IL16 | Pro-interleukin-16 | Q14005 | *IL16* | 3603 | -0.097 | -0.234 | 0.036 | 0.1545 | 0.6400 |
| PLC | Perlecan | P98160 | *HSPG2* | 3339 | 0.049 | -0.019 | 0.119 | 0.1593 | 0.6400 |
| OSM | Oncostatin-M | P13725 | *OSM* | 5008 | -0.143 | -0.340 | 0.057 | 0.1627 | 0.6400 |
| CCL15 | C-C motif chemokine 15 | Q16663 | *CCL15* | 6359 | 0.055 | -0.022 | 0.132 | 0.1639 | 0.6400 |
| PAR-1 | Proteinase-activated receptor 1 | P25116 | *F2R* | 2149 | -0.111 | -0.270 | 0.047 | 0.1652 | 0.6400 |
| ITGB1BP2 | Melusin | Q9UKP3 | *ITGB1BP2* | 26548 | -0.152 | -0.373 | 0.065 | 0.1672 | 0.6400 |
| OSMR | Oncostatin-M-specific receptor subunit beta | Q99650 | *OSMR* | 9180 | -0.033 | -0.082 | 0.015 | 0.1677 | 0.6400 |
| PCSK9 | Proprotein convertase subtilisin/kexin type 9 | Q8NBP7 | *PCSK9* | 255738 | 0.063 | -0.028 | 0.149 | 0.1698 | 0.6400 |
| Flt3L | Fms-related tyrosine kinase 3 ligand | P49771 | *FLT3LG* | 2323 | -0.070 | -0.170 | 0.030 | 0.1723 | 0.6400 |
| vWF | von Willebrand factor | P04275 | *VWF* | 7450 | -0.117 | -0.295 | 0.055 | 0.1761 | 0.6400 |
| ANG-1 | Angiopoietin-1 | Q15389 | *ANGPT1* | 284 | -0.165 | -0.401 | 0.072 | 0.1770 | 0.6400 |
| CXCL10 | C-X-C motif chemokine 10 | P02778 | *CXCL10* | 3627 | -0.098 | -0.244 | 0.045 | 0.1774 | 0.6400 |
| FABP4 | Fatty acid-binding protein, adipocyte | P15090 | *FABP4* | 2167 | -0.099 | -0.254 | 0.050 | 0.1774 | 0.6400 |
| MPO | Myeloperoxidase | P05164 | *MPO* | 4353 | -0.060 | -0.143 | 0.025 | 0.1776 | 0.6400 |
| SELE | E-selectin | P16581 | *SELE* | 6401 | 0.096 | -0.045 | 0.234 | 0.1796 | 0.6400 |
| ALCAM | CD166 antigen | Q13740 | *ALCAM* | 214 | -0.040 | -0.099 | 0.019 | 0.1820 | 0.6400 |
| ACE2 | Angiotensin-converting enzyme 2 | Q9BYF1 | *ACE2* | 59272 | 0.082 | -0.038 | 0.194 | 0.1843 | 0.6400 |
| CCL23 | C-C motif chemokine 23 | P55773 | *CCL23* | 6368 | -0.069 | -0.172 | 0.032 | 0.1880 | 0.6400 |
| TNFB | TNF-beta | P01374 | *LTA* | 4049 | -0.066 | -0.166 | 0.033 | 0.1890 | 0.6400 |
| HO-1 | Heme oxygenase 1 | P09601 | *HMOX1* | 3162 | 0.095 | -0.045 | 0.230 | 0.1894 | 0.6400 |
| CD163 | Scavenger receptor cysteine-rich type 1 protein M130 | Q86VB7 | *CD163* | 9332 | -0.072 | -0.174 | 0.036 | 0.1898 | 0.6400 |
| MET | Hepatocyte growth factor receptor | P08581 | *MET* | 4233 | -0.031 | -0.078 | 0.016 | 0.1898 | 0.6400 |
| STK4 | Serine/threonine-protein kinase 4 | Q13043 | *STK4* | 6789 | -0.192 | -0.490 | 0.097 | 0.1898 | 0.6400 |
| GP6 | Platelet glycoprotein VI | Q9HCN6 | *GP6* | 51206 | -0.096 | -0.245 | 0.052 | 0.1996 | 0.6519 |
| SPARCL1 | SPARC-like protein 1 | Q14515 | *SPARCL1* | 8404 | -0.036 | -0.091 | 0.020 | 0.1997 | 0.6519 |
| ST1A1 | Sulfotransferase 1A1 | P50225 | *SULT1A1* | 6817 | -0.213 | -0.544 | 0.111 | 0.2005 | 0.6519 |
| CA1 | Carbonic anhydrase 1 | P00915 | *CA1* | 759 | -0.119 | -0.301 | 0.061 | 0.2007 | 0.6519 |
| IL-22 RA1 | Interleukin-22 receptor subunit alpha-1 | Q8N6P7 | *IL22RA1* | 58985 | -0.067 | -0.167 | 0.036 | 0.2032 | 0.6541 |
| CD84 | SLAM family member 5 | Q9UIB8 | *CD84* | 8832 | -0.080 | -0.206 | 0.045 | 0.2058 | 0.6562 |
| ICAM-2 | Intercellular adhesion molecule 2 | P13598 | *ICAM2* | 3384 | -0.055 | -0.143 | 0.031 | 0.2092 | 0.6606 |
| IL-33 | Interleukin-33 | O95760 | *IL33* | 90865 | 0.036 | -0.021 | 0.092 | 0.2109 | 0.6606 |
| AP-N | Aminopeptidase N | P15144 | *ANPEP* | 290 | 0.042 | -0.025 | 0.106 | 0.2168 | 0.6733 |
| MFAP5 | Microfibrillar-associated protein 5 | Q13361 | *MFAP5* | 8076 | -0.034 | -0.088 | 0.019 | 0.2194 | 0.6752 |
| SERPINA12 | Serpin A12 | Q8IW75 | *SERPINA12* | 145264 | -0.149 | -0.397 | 0.092 | 0.2216 | 0.6764 |
| IL10 | Interleukin-10 | P22301 | *IL10* | 3586 | -0.057 | -0.150 | 0.035 | 0.2242 | 0.6781 |
| UMOD | Uromodulin | P07911 | *UMOD* | 7369 | -0.038 | -0.097 | 0.024 | 0.2300 | 0.6781 |
| IL-20 | Interleukin-20 | Q9NYY1 | *IL20* | 50604 | -0.030 | -0.080 | 0.020 | 0.2329 | 0.6781 |
| PD-L2 | Programmed cell death 1 ligand 2 | Q9BQ51 | *PDCD1LG2* | 80380 | -0.052 | -0.135 | 0.032 | 0.2330 | 0.6781 |
| C1QTNF1 | Complement C1q tumor necrosis factor-related protein 1 | Q9BXJ1 | *C1QTNF1* | 114897 | 0.057 | -0.039 | 0.149 | 0.2334 | 0.6781 |
| VEGF-A | Vascular endothelial growth factor A | P15692 | *VEGFA* | 7422 | -0.073 | -0.195 | 0.047 | 0.2350 | 0.6781 |
| TNC | Tenascin | P24821 | *TNC* | 3371 | -0.060 | -0.160 | 0.039 | 0.2373 | 0.6781 |
| IL7R | Interleukin-7 receptor subunit alpha | P16871 | *IL7R* | 3575 | 0.062 | -0.041 | 0.166 | 0.2384 | 0.6781 |
| C2 | Complement C2 | P06681 | *C2* | 717 | -0.095 | -0.259 | 0.063 | 0.2394 | 0.6781 |
| Gal-4 | Galectin-4 | P56470 | *LGALS4* | 3960 | 0.056 | -0.039 | 0.148 | 0.2451 | 0.6887 |
| THBS4 | Thrombospondin-4 | P35443 | *THBS4* | 7060 | -0.070 | -0.195 | 0.054 | 0.2587 | 0.6986 |
| CDH5 | Cadherin-5 | P33151 | *CDH5* | 1003 | 0.048 | -0.035 | 0.129 | 0.2588 | 0.6986 |
| CA4 | Carbonic anhydrase 4 | P22748 | *CA4* | 762 | -0.033 | -0.088 | 0.025 | 0.2592 | 0.6986 |
| MMP-1 | Matrix metalloproteinase-1 | P03956 | *MMP1* | 4312 | -0.185 | -0.520 | 0.150 | 0.2648 | 0.6986 |
| CD40-L | CD40 ligand | P29965 | *CD40LG* | 959 | -0.175 | -0.483 | 0.129 | 0.2657 | 0.6986 |
| LDL receptor | Low-density lipoprotein receptor | P01130 | *LDLR* | 3949 | 0.077 | -0.053 | 0.203 | 0.2663 | 0.6986 |
| TR | Transferrin receptor protein 1 | P02786 | *TFRC* | 7037 | -0.052 | -0.145 | 0.041 | 0.2690 | 0.6986 |
| CCL5 | C-C motif chemokine 5 | P13501 | *CCL5* | 6352 | -0.132 | -0.381 | 0.113 | 0.2697 | 0.6986 |
| IGFBP3 | Insulin-like growth factor-binding protein 3 | P17936 | *IGFBP3* | 3486 | -0.047 | -0.128 | 0.037 | 0.2699 | 0.6986 |
| DCN | Decorin | P07585 | *DCN* | 1634 | -0.036 | -0.099 | 0.026 | 0.2708 | 0.6986 |
| PLXNB2 | Plexin-B2 | O15031 | *PLXNB2* | 23654 | -0.025 | -0.072 | 0.021 | 0.2737 | 0.6986 |
| TIMD4 | T-cell immunoglobulin and mucin domain-containing protein 4 | Q96H15 | *TIMD4* | 91937 | -0.051 | -0.147 | 0.043 | 0.2745 | 0.6986 |
| MCP-1 | Monocyte chemotactic protein 1 | P13500 | *CCL2* | 6347 | -0.056 | -0.157 | 0.046 | 0.2771 | 0.6986 |
| CD4 | T-cell surface glycoprotein CD4 | P01730 | *CD4* | 920 | 0.046 | -0.038 | 0.127 | 0.2783 | 0.6986 |
| CTSL1 | Cathepsin L1 | P07711 | *CTSL* | 1514 | 0.060 | -0.049 | 0.168 | 0.2806 | 0.6986 |
| COL18A1 | Collagen alpha-1(XVIII) chain | P39060 | *COL18A1* | 80781 | -0.039 | -0.109 | 0.032 | 0.2846 | 0.6986 |
| NT-3 | Neurotrophin-3 | P20783 | *NTF3* | 4908 | -0.042 | -0.120 | 0.036 | 0.2846 | 0.6986 |
| IL1RL2 | Interleukin-1 receptor-like 2 | Q9HB29 | *IL1RL2* | 8808 | -0.049 | -0.139 | 0.042 | 0.2858 | 0.6986 |
| IGFBP-2 | Insulin-like growth factor-binding protein 2 | P18065 | *IGFBP2* | 3485 | -0.086 | -0.247 | 0.075 | 0.2862 | 0.6986 |
| CCL17 | C-C motif chemokine 17 | Q92583 | *CCL17* | 6361 | -0.115 | -0.330 | 0.098 | 0.2929 | 0.7102 |
| PCOLCE | Procollagen C-endopeptidase enhancer 1 | Q15113 | *PCOLCE* | 5118 | 0.048 | -0.042 | 0.138 | 0.2977 | 0.7114 |
| AXIN1 | Axin-1 | O15169 | *AXIN1* | 8312 | -0.124 | -0.363 | 0.109 | 0.2988 | 0.7114 |
| U-PAR | Urokinase plasminogen activator surface receptor | Q03405 | *PLAUR* | 5329 | -0.043 | -0.133 | 0.042 | 0.3009 | 0.7114 |
| AZU1 | Azurocidin | P20160 | *AZU1* | 566 | -0.070 | -0.215 | 0.066 | 0.3061 | 0.7114 |
| KIT | Mast/stem cell growth factor receptor Kit | P10721 | *KIT* | 3815 | -0.044 | -0.130 | 0.039 | 0.3075 | 0.7114 |
| EN-RAGE | Protein S100-A12 | P80511 | *S100A12* | 6283 | -0.096 | -0.282 | 0.090 | 0.3093 | 0.7114 |
| TIMP1 | Metalloproteinase inhibitor 1 | P01033 | *TIMP1* | 7076 | -0.035 | -0.098 | 0.030 | 0.3120 | 0.7114 |
| CD46 | Membrane cofactor protein | P15529 | *CD46* | 4179 | -0.032 | -0.096 | 0.030 | 0.3135 | 0.7114 |
| MEPE | Matrix extracellular phosphoglycoprotein | Q9NQ76 | *MEPE* | 56955 | 0.072 | -0.072 | 0.215 | 0.3136 | 0.7114 |
| MMP-12 | Matrix metalloproteinase-12 | P39900 | *MMP12* | 4321 | -0.074 | -0.218 | 0.071 | 0.3160 | 0.7114 |
| PGF | Placenta growth factor | P49763 | *PGF* | 5228 | -0.037 | -0.109 | 0.035 | 0.3170 | 0.7114 |
| SOD2 | Superoxide dismutase [Mn], mitochondrial | P04179 | *SOD2* | 6648 | -0.039 | -0.117 | 0.038 | 0.3196 | 0.7114 |
| GDNF | Glial cell line-derived neurotrophic factor | P39905 | *GDNF* | 2668 | -0.039 | -0.114 | 0.040 | 0.3233 | 0.7114 |
| CD40 | CD40L receptor | P25942 | *CD40* | 958 | -0.053 | -0.158 | 0.053 | 0.3256 | 0.7114 |
| PDGF subunit A | Platelet-derived growth factor subunit A | P04085 | *PDGFA* | 5154 | -0.104 | -0.313 | 0.100 | 0.3256 | 0.7114 |
| GLO1 | Lactoylglutathione lyase | Q04760 | *GLO1* | 2739 | -0.101 | -0.304 | 0.101 | 0.3259 | 0.7114 |
| GRN | Granulins | P28799 | *GRN* | 2896 | -0.033 | -0.103 | 0.034 | 0.3276 | 0.7114 |
| NID1 | Nidogen-1 | P14543 | *NID1* | 4811 | -0.040 | -0.126 | 0.043 | 0.3339 | 0.7208 |
| PSP-D | Pulmonary surfactant-associated protein D | P35247 | *SFTPD* | 6441 | 0.071 | -0.072 | 0.211 | 0.3383 | 0.7259 |
| CRTAC1 | Cartilage acidic protein 1 | Q9NQ79 | *CRTAC1* | 55118 | -0.047 | -0.145 | 0.051 | 0.3452 | 0.7350 |
| TGFBR3 | Transforming growth factor beta receptor type 3 | Q03167 | *TGFBR3* | 7049 | -0.032 | -0.098 | 0.033 | 0.3486 | 0.7350 |
| TRANCE | TNF-related activation-induced cytokine | O14788 | *TNFSF11* | 8600 | -0.069 | -0.206 | 0.073 | 0.3488 | 0.7350 |
| IL6 | Interleukin-6 | P05231 | *IL6* | 3569 | -0.058 | -0.192 | 0.070 | 0.3531 | 0.7366 |
| LILRB1 | Leukocyte immunoglobulin-like receptor subfamily B member 1 | Q8NHL6 | *LILRB1* | 10859 | -0.025 | -0.081 | 0.029 | 0.3556 | 0.7366 |
| ARTN | Artemin | Q5T4W7 | *ARTN* | 9048 | -0.022 | -0.072 | 0.026 | 0.3570 | 0.7366 |
| CA3 | Carbonic anhydrase 3 | P07451 | *CA3* | 761 | -0.025 | -0.080 | 0.027 | 0.3579 | 0.7366 |
| SLAMF1 | Signaling lymphocytic activation molecule | Q13291 | *SLAMF1* | 6504 | -0.037 | -0.117 | 0.044 | 0.3655 | 0.7479 |
| CHI3L1 | Chitinase-3-like protein 1 | P36222 | *CHI3L1* | 1116 | -0.068 | -0.238 | 0.092 | 0.3684 | 0.7494 |
| ANG | Angiogenin | P03950 | *ANG* | 283 | 0.037 | -0.048 | 0.128 | 0.3714 | 0.7513 |
| CSTB | Cystatin-B | P04080 | *CSTB* | 1476 | -0.049 | -0.162 | 0.061 | 0.3788 | 0.7537 |
| CASP-3 | Caspase-3 | P42574 | *CASP3* | 836 | -0.120 | -0.394 | 0.149 | 0.3796 | 0.7537 |
| VEGFD | Vascular endothelial growth factor D | O43915 | *VEGFD* | 2277 | -0.045 | -0.148 | 0.052 | 0.3816 | 0.7537 |
| DLK-1 | Protein delta homolog 1 | P80370 | *DLK1* | 8788 | 0.059 | -0.081 | 0.196 | 0.3832 | 0.7537 |
| CD8A | T-cell surface glycoprotein CD8 alpha chain | P01732 | *CD8A* | 925 | -0.093 | -0.306 | 0.119 | 0.3832 | 0.7537 |
| t-PA | Tissue-type plasminogen activator | P00750 | *PLAT* | 5327 | 0.061 | -0.077 | 0.200 | 0.3918 | 0.7662 |
| AXL | Tyrosine-protein kinase receptor UFO | P30530 | *AXL* | 558 | 0.031 | -0.042 | 0.102 | 0.3959 | 0.7700 |
| ADA | Adenosine Deaminase | P00813 | *ADA* | 100 | -0.050 | -0.167 | 0.065 | 0.4030 | 0.7726 |
| TNF | Tumor necrosis factor | P01375 | *TNF* | 7124 | -0.025 | -0.084 | 0.032 | 0.4036 | 0.7726 |
| hOSCAR | Osteoclast-associated immunoglobulin-like receptor | Q8IYS5 | *OSCAR* | 126014 | -0.029 | -0.096 | 0.038 | 0.4038 | 0.7726 |
| RARRES2 | Retinoic acid receptor responder protein 2 | Q99969 | *RARRES2* | 5919 | -0.027 | -0.095 | 0.040 | 0.4191 | 0.7739 |
| ENG | Endoglin | P17813 | *ENG* | 2022 | -0.023 | -0.077 | 0.030 | 0.4211 | 0.7739 |
| CCL25 | C-C motif chemokine 25 | O15444 | *CCL25* | 6370 | -0.062 | -0.215 | 0.089 | 0.4238 | 0.7739 |
| THBS2 | Thrombospondin-2 | P35442 | *THBS2* | 7058 | 0.019 | -0.029 | 0.070 | 0.4283 | 0.7739 |
| TNFRSF14 | Tumor necrosis factor receptor superfamily member 14 | Q92956 | *TNFRSF14* | 8764 | -0.027 | -0.094 | 0.040 | 0.4289 | 0.7739 |
| VASN | Vasorin | Q6EMK4 | *VASN* | 114990 | -0.023 | -0.081 | 0.035 | 0.4293 | 0.7739 |
| CR2 | Complement receptor type 2 | P20023 | *CR2* | 1380 | -0.040 | -0.142 | 0.062 | 0.4297 | 0.7739 |
| LAP TGF-beta-1 | Latency-associated peptide transforming growth factor beta-1 | P01137 | *TGFB1* | 7040 | -0.057 | -0.202 | 0.086 | 0.4299 | 0.7739 |
| MARCO | Macrophage receptor MARCO | Q9UEW3 | *MARCO* | 8685 | -0.033 | -0.117 | 0.048 | 0.4352 | 0.7739 |
| TWEAK | Tumor necrosis factor (Ligand) superfamily, member 12 | O43508 | *TNFSF12* | 8742 | -0.044 | -0.157 | 0.069 | 0.4358 | 0.7739 |
| PAPPA | Pappalysin-1 | Q13219 | *PAPPA* | 5069 | -0.032 | -0.113 | 0.049 | 0.4364 | 0.7739 |
| ICAM1 | Intercellular adhesion molecule 1 | P05362 | *ICAM1* | 3383 | -0.030 | -0.109 | 0.049 | 0.4372 | 0.7739 |
| TNF-R1 | Tumor necrosis factor receptor 1 | P19438 | *TNFRSF1A* | 7132 | -0.029 | -0.100 | 0.048 | 0.4378 | 0.7739 |
| LIF | Leukemia inhibitory factor | P15018 | *LIF* | 3976 | -0.024 | -0.084 | 0.035 | 0.4400 | 0.7739 |
| ST2 | ST2 protein | Q01638 | *IL1RL1* | 9173 | 0.039 | -0.065 | 0.141 | 0.4420 | 0.7739 |
| TNF-R2 | Tumor necrosis factor receptor 2 | P20333 | *TNFRSF1B* | 7133 | -0.029 | -0.103 | 0.044 | 0.4420 | 0.7739 |
| DECR1 | 2,4-dienoyl-CoA reductase, mitochondrial | Q16698 | *DECR1* | 1666 | -0.157 | -0.573 | 0.263 | 0.4420 | 0.7739 |
| CCL24 | C-C motif chemokine 24 | O00175 | *CCL24* | 6369 | 0.091 | -0.136 | 0.314 | 0.4448 | 0.7739 |
| MEGF9 | Multiple epidermal growth factor-like domains protein 9 | Q9H1U4 | *MEGF9* | 1955 | -0.025 | -0.093 | 0.042 | 0.4464 | 0.7739 |
| NCAM1 | Neural cell adhesion molecule 1 | P13591 | *NCAM1* | 4684 | -0.025 | -0.090 | 0.038 | 0.4512 | 0.7739 |
| CNDP1 | Beta-Ala-His dipeptidase | Q96KN2 | *CNDP1* | 84735 | 0.052 | -0.086 | 0.187 | 0.4559 | 0.7739 |
| FGF-19 | Fibroblast growth factor 19 | O95750 | *FGF19* | 9965 | -0.078 | -0.284 | 0.128 | 0.4581 | 0.7739 |
| XCL1 | Lymphotactin | P47992 | *XCL1* | 6375 | 0.064 | -0.107 | 0.229 | 0.4594 | 0.7739 |
| DPP4 | Dipeptidyl peptidase 4 | P27487 | *DPP4* | 1803 | -0.031 | -0.115 | 0.050 | 0.4600 | 0.7739 |
| CNTN1 | Contactin-1 | Q12860 | *CNTN1* | 1272 | -0.028 | -0.101 | 0.046 | 0.4630 | 0.7739 |
| CXCL16 | C-X-C motif chemokine 16 | Q9H2A7 | *CXCL16* | 58191 | 0.023 | -0.040 | 0.089 | 0.4632 | 0.7739 |
| PARP-1 | Poly [ADP-ribose] polymerase 1 | P09874 | *PARP1* | 142 | -0.060 | -0.221 | 0.110 | 0.4634 | 0.7739 |
| IL-1RT2 | Interleukin-1 receptor type 2 | P27930 | *IL1R2* | 7850 | 0.024 | -0.042 | 0.091 | 0.4692 | 0.7742 |
| SELP | P-selectin | P16109 | *SELP* | 6403 | -0.062 | -0.221 | 0.102 | 0.4696 | 0.7742 |
| IL-10RA | Interleukin-10 receptor subunit alpha | Q13651 | *IL10RA* | 3587 | 0.029 | -0.047 | 0.109 | 0.4717 | 0.7742 |
| CTSD | Cathepsin D | P07339 | *CTSD* | 1509 | 0.031 | -0.056 | 0.119 | 0.4767 | 0.7742 |
| TNXB | Tenascin-X | P22105 | *TNXB* | 7148 | -0.015 | -0.056 | 0.027 | 0.4817 | 0.7742 |
| TNFRSF11A | Tumor necrosis factor receptor superfamily member 11A | Q9Y6Q6 | *TNFRSF11A* | 8792 | -0.035 | -0.132 | 0.062 | 0.4826 | 0.7742 |
| CPA1 | Carboxypeptidase A1 | P15085 | *CPA1* | 1357 | 0.045 | -0.086 | 0.182 | 0.4830 | 0.7742 |
| LPL | Lipoprotein lipase | P06858 | *LPL* | 4023 | 0.063 | -0.113 | 0.239 | 0.4868 | 0.7742 |
| IL-4 | Interleukin-4 | P05112 | *IL4* | 3565 | -0.029 | -0.115 | 0.054 | 0.4901 | 0.7742 |
| PSGL-1 | P-selectin glycoprotein ligand 1 | Q14242 | *SELPLG* | 6404 | 0.021 | -0.038 | 0.081 | 0.4914 | 0.7742 |
| THPO | Thrombopoietin | P40225 | *THPO* | 7066 | -0.023 | -0.090 | 0.043 | 0.4944 | 0.7742 |
| SHPS-1 | Tyrosine-protein phosphatase non-receptor type substrate 1 | P78324 | *SIRPA* | 140885 | -0.029 | -0.114 | 0.053 | 0.4957 | 0.7742 |
| CA5A | Carbonic anhydrase 5A, mitochondrial | P35218 | *CA5A* | 763 | 0.051 | -0.093 | 0.194 | 0.4957 | 0.7742 |
| TRAIL-R2 | TNF-related apoptosis-inducing ligand receptor 2 | O14763 | *TNFRSF10B* | 8795 | -0.026 | -0.104 | 0.052 | 0.4967 | 0.7742 |
| GP1BA | Platelet glycoprotein Ib alpha chain | P07359 | *GP1BA* | 2811 | -0.040 | -0.153 | 0.074 | 0.4976 | 0.7742 |
| IL-12B | Interleukin-12 subunit beta | P29460 | *IL12B* | 3593 | 0.046 | -0.093 | 0.176 | 0.4987 | 0.7742 |
| Notch 3 | Neurogenic locus notch homolog protein 3 | Q9UM47 | *NOTCH3* | 4854 | -0.027 | -0.111 | 0.056 | 0.5025 | 0.7768 |
| IL-15RA | Interleukin-15 receptor subunit alpha | Q13261 | *IL15RA* | 3601 | -0.014 | -0.058 | 0.029 | 0.5092 | 0.7837 |
| TM | Thrombomodulin TM | P07204 | *THBD* | 7056 | -0.026 | -0.106 | 0.054 | 0.5211 | 0.7986 |
| AOC3 | Membrane primary amine oxidase | Q16853 | *AOC3* | 8639 | -0.026 | -0.109 | 0.054 | 0.5247 | 0.8006 |
| ST6GAL1 | Beta-galactoside alpha-2,6-sialyltransferase 1 | P15907 | *ST6GAL1* | 6480 | -0.022 | -0.094 | 0.047 | 0.5308 | 0.8065 |
| FGF-21 | Fibroblast growth factor 21 | Q9NSA1 | *FGF21* | 26291 | 0.091 | -0.202 | 0.396 | 0.5348 | 0.8075 |
| CST5 | Cystatin D | P28325 | *CST5* | 1473 | 0.039 | -0.088 | 0.166 | 0.5370 | 0.8075 |
| CES1 | Liver carboxylesterase 1 | P23141 | *CES1* | 1066 | 0.035 | -0.077 | 0.148 | 0.5384 | 0.8075 |
| AMBP | Protein AMBP | P02760 | *AMBP* | 259 | 0.016 | -0.039 | 0.071 | 0.5554 | 0.8240 |
| DNER | Delta and Notch-like epidermal growth factor-related receptor | Q8NFT8 | *DNER* | 92737 | -0.018 | -0.077 | 0.043 | 0.5579 | 0.8240 |
| IGFBP-1 | Insulin-like growth factor-binding protein 1 | P08833 | *IGFBP1* | 3484 | -0.067 | -0.305 | 0.171 | 0.5608 | 0.8240 |
| IGLC2 | Ig lambda-2 chain C regions | P0CG05 | *IGLC2* | 3538 | -0.031 | -0.131 | 0.076 | 0.5617 | 0.8240 |
| TNFSF13B | Tumor necrosis factor ligand superfamily member 13B | Q9Y275 | *TNFSF13B* | 10673 | -0.020 | -0.091 | 0.050 | 0.5631 | 0.8240 |
| COMP | Cartilage oligomeric matrix protein | P49747 | *COMP* | 1311 | -0.026 | -0.118 | 0.066 | 0.5647 | 0.8240 |
| CD5 | T-cell surface glycoprotein CD5 | P06127 | *CD5* | 921 | -0.022 | -0.097 | 0.055 | 0.5658 | 0.8240 |
| MERTK | Tyrosine-protein kinase Mer | Q12866 | *MERTK* | 10461 | 0.025 | -0.063 | 0.112 | 0.5704 | 0.8240 |
| TFF3 | Trefoil factor 3 | Q07654 | *TFF3* | 7033 | -0.021 | -0.101 | 0.056 | 0.5706 | 0.8240 |
| TGFBI | Transforming growth factor-beta-induced protein ig-h3 | Q15582 | *TGFBI* | 7045 | -0.025 | -0.114 | 0.067 | 0.5741 | 0.8240 |
| MMP-10 | Matrix metalloproteinase-10 | P09238 | *MMP10* | 4319 | 0.040 | -0.104 | 0.183 | 0.5754 | 0.8240 |
| GIF | Gastric intrinsic factor | P27352 | *CBLIF* | 2694 | 0.052 | -0.138 | 0.238 | 0.5773 | 0.8240 |
| TGM2 | Protein-glutamine gamma-glutamyltransferase 2 | P21980 | *TGM2* | 7052 | -0.033 | -0.154 | 0.088 | 0.5805 | 0.8253 |
| BNP | Natriuretic peptides B | P16860 | *NPPB* | 4879 | -0.011 | -0.052 | 0.028 | 0.5837 | 0.8266 |
| MMP-9 | Matrix metalloproteinase-9 | P14780 | *MMP9* | 4318 | -0.049 | -0.223 | 0.119 | 0.5898 | 0.8318 |
| ICAM3 | Intercellular adhesion molecule 3 | P32942 | *ICAM3* | 3385 | -0.017 | -0.082 | 0.046 | 0.5935 | 0.8337 |
| SPON1 | Spondin-1 | Q9HCB6 | *SPON1* | 10418 | 0.016 | -0.043 | 0.073 | 0.5979 | 0.8366 |
| IL5 | Interleukin-5 | P05113 | *IL5* | 3567 | 0.030 | -0.082 | 0.141 | 0.6104 | 0.8480 |
| NRP1 | Neuropilin-1 | O14786 | *NRP1* | 8829 | -0.009 | -0.044 | 0.025 | 0.6108 | 0.8480 |
| CCL16 | C-C motif chemokine 16 | O15467 | *CCL16* | 6360 | -0.027 | -0.138 | 0.082 | 0.6146 | 0.8491 |
| F11 | Coagulation factor XI | P03951 | *F11* | 2160 | -0.017 | -0.081 | 0.050 | 0.6203 | 0.8491 |
| VSIG2 | V-set and immunoglobulin domain-containing protein 2 | Q96IQ7 | *VSIG2* | 23584 | -0.024 | -0.120 | 0.075 | 0.6222 | 0.8491 |
| EFEMP1 | EGF-containing fibulin-like extracellular matrix protein 1 | Q12805 | *EFEMP1* | 2202 | 0.033 | -0.103 | 0.169 | 0.6236 | 0.8491 |
| CCL11 | Eotaxin | P51671 | *CCL11* | 6356 | -0.029 | -0.154 | 0.095 | 0.6236 | 0.8491 |
| IL-17A | Interleukin-17A | Q16552 | *IL17A* | 3605 | -0.017 | -0.088 | 0.054 | 0.6265 | 0.8497 |
| ADM | ADM | P35318 | *ADM* | 133 | 0.105 | -0.324 | 0.546 | 0.6327 | 0.8501 |
| GH | Growth hormone | P01241 | *GH1* | 2688 | -0.132 | -0.687 | 0.398 | 0.6342 | 0.8501 |
| TIMP4 | Metalloproteinase inhibitor 4 | Q99727 | *TIMP4* | 7079 | -0.026 | -0.128 | 0.077 | 0.6342 | 0.8501 |
| IL-17D | Interleukin-17D | Q8TAD2 | *IL17D* | 53342 | -0.014 | -0.075 | 0.045 | 0.6385 | 0.8501 |
| SERPINA5 | Plasma serine protease inhibitor | P05154 | *SERPINA5* | 5104 | 0.018 | -0.061 | 0.092 | 0.6390 | 0.8501 |
| CD6 | T cell surface glycoprotein CD6 isoform | Q8WWJ7 | *CD6* | 923 | -0.025 | -0.132 | 0.083 | 0.6433 | 0.8501 |
| PAM | Peptidyl-glycine alpha-amidating monooxygenase | P19021 | *PAM* | 5066 | -0.021 | -0.116 | 0.071 | 0.6435 | 0.8501 |
| PLTP | Phospholipid transfer protein | P55058 | *PLTP* | 5360 | -0.012 | -0.061 | 0.039 | 0.6472 | 0.8517 |
| NRTN | Neurturin | Q99748 | *NRTN* | 4902 | -0.012 | -0.072 | 0.041 | 0.6696 | 0.8779 |
| MB | Myoglobin | P02144 | *MB* | 4151 | 0.025 | -0.091 | 0.140 | 0.6745 | 0.8811 |
| MMP-7 | Matrix metalloproteinase-7 | P09237 | *MMP7* | 4316 | 0.089 | -0.319 | 0.473 | 0.6789 | 0.8836 |
| FS | Follistatin | P19883 | *FST* | 10468 | 0.030 | -0.105 | 0.166 | 0.6908 | 0.8938 |
| Protein BOC | Brother of CDO | Q9BWV1 | *BOC* | 91653 | -0.014 | -0.088 | 0.058 | 0.6918 | 0.8938 |
| CDCP1 | CUB domain-containing protein 1 | Q9H5V8 | *CDCP1* | 64866 | -0.025 | -0.159 | 0.104 | 0.6998 | 0.9008 |
| TRAIL | TNF-related apoptosis-inducing ligand | P50591 | *TNFSF10* | 8743 | -0.018 | -0.115 | 0.076 | 0.7028 | 0.9014 |
| MCP-3 | Monocyte chemotactic protein 3 | P80098 | *CCL7* | 6354 | -0.018 | -0.112 | 0.079 | 0.7103 | 0.9056 |
| PRSS2 | Trypsin-2 | P07478 | *PRSS2* | 5645 | -0.018 | -0.106 | 0.072 | 0.7153 | 0.9056 |
| 4E-BP1 | Eukaryotic translation initiation factor 4E-binding protein 1 | Q13541 | *EIF4EBP1* | 1978 | -0.062 | -0.390 | 0.275 | 0.7163 | 0.9056 |
| IL-24 | Interleukin-24 | Q13007 | *IL24* | 11009 | 0.015 | -0.076 | 0.107 | 0.7163 | 0.9056 |
| ADAM-TS13 | A disintegrin and metalloproteinase with thrombospondin motifs 13 | Q76LX8 | *ADAMTS13* | 11093 | -0.008 | -0.055 | 0.040 | 0.7284 | 0.9134 |
| Gal-3 | Galectin-3 | P17931 | *LGALS3* | 3958 | -0.013 | -0.087 | 0.061 | 0.7289 | 0.9134 |
| APOM | Apolipoprotein M | O95445 | *APOM* | 55937 | -0.016 | -0.108 | 0.072 | 0.7302 | 0.9134 |
| SCGB3A2 | Secretoglobin family 3A member 2 | Q96PL1 | *SCGB3A2* | 117156 | -0.030 | -0.202 | 0.150 | 0.7426 | 0.9185 |
| MBL2 | Mannose-binding protein C | P11226 | *MBL2* | 4153 | -0.044 | -0.315 | 0.236 | 0.7431 | 0.9185 |
| IL-17RA | Interleukin-17 receptor A | Q96F46 | *IL17RA* | 23765 | -0.019 | -0.133 | 0.096 | 0.7442 | 0.9185 |
| CDH1 | Cadherin-1 | P12830 | *CDH1* | 999 | -0.013 | -0.093 | 0.068 | 0.7454 | 0.9185 |
| LILRB5 | Leukocyte immunoglobulin-like receptor subfamily B member 5 | O75023 | *LILRB5* | 10990 | -0.026 | -0.180 | 0.125 | 0.7472 | 0.9185 |
| FCGR2A | Low affinity immunoglobulin gamma Fc region receptor II-a | P12318 | *FCGR2A* | 2212 | 0.019 | -0.104 | 0.142 | 0.7511 | 0.9200 |
| FAP | Prolyl endopeptidase FAP | Q12884 | *FAP* | 2191 | -0.008 | -0.061 | 0.047 | 0.7600 | 0.9277 |
| SPON2 | Spondin-2 | Q9BUD6 | *SPON2* | 10417 | 0.011 | -0.055 | 0.078 | 0.7636 | 0.9289 |
| JAM-A | Junctional adhesion molecule A | Q9Y624 | *F11R* | 50848 | 0.030 | -0.167 | 0.238 | 0.7662 | 0.9289 |
| PGLYRP1 | Peptidoglycan recognition protein 1 | O75594 | *PGLYRP1* | 8993 | 0.020 | -0.122 | 0.162 | 0.7868 | 0.9336 |
| FABP2 | Fatty acid-binding protein, intestinal | P12104 | *FABP2* | 2169 | -0.021 | -0.189 | 0.139 | 0.7873 | 0.9336 |
| CD59 | CD59 glycoprotein | P13987 | *CD59* | 966 | -0.007 | -0.059 | 0.043 | 0.7897 | 0.9336 |
| IL-18BP | Interleukin-18-binding protein | O95998 | *IL18BP* | 10068 | -0.009 | -0.083 | 0.062 | 0.7910 | 0.9336 |
| TIE2 | Angiopoietin-1 receptor | Q02763 | *TEK* | 7010 | 0.008 | -0.050 | 0.065 | 0.7936 | 0.9336 |
| RAGE | Receptor for advanced glycosylation end products | Q15109 | *AGER* | 177 | -0.011 | -0.089 | 0.070 | 0.7977 | 0.9336 |
| CTSZ | Cathepsin Z | Q9UBR2 | *CTSZ* | 1522 | -0.010 | -0.088 | 0.068 | 0.7977 | 0.9336 |
| IL-1RT1 | Interleukin-1 receptor type 1 | P14778 | *IL1R1* | 3554 | -0.008 | -0.068 | 0.051 | 0.7998 | 0.9336 |
| CCL28 | C-C motif chemokine 28 | Q9NRJ3 | *CCL28* | 56477 | 0.010 | -0.066 | 0.087 | 0.8035 | 0.9336 |
| IGFBP-7 | Insulin-like growth factor-binding protein 7 | Q16270 | *IGFBP7* | 3490 | 0.009 | -0.060 | 0.078 | 0.8040 | 0.9336 |
| PIgR | Polymeric immunoglobulin receptor | P01833 | *PIGR* | 5284 | 0.007 | -0.047 | 0.057 | 0.8055 | 0.9336 |
| F7 | Coagulation factor VII | P08709 | *F7* | 2155 | -0.010 | -0.089 | 0.068 | 0.8071 | 0.9336 |
| SAA4 | Serum amyloid A-4 protein | P35542 | *SAA4* | 6291 | 0.020 | -0.138 | 0.188 | 0.8087 | 0.9336 |
| IL-17C | Interleukin-17C | Q9P0M4 | *IL17C* | 27189 | -0.009 | -0.080 | 0.060 | 0.8087 | 0.9336 |
| OPN | Osteopontin | P10451 | *SPP1* | 6696 | 0.021 | -0.138 | 0.180 | 0.8118 | 0.9336 |
| LTBR | Lymphotoxin-beta receptor | P36941 | *LTBR* | 4055 | -0.007 | -0.068 | 0.056 | 0.8128 | 0.9336 |
| EPHB4 | Ephrin type-B receptor 4 | P54760 | *EPHB4* | 2050 | -0.007 | -0.066 | 0.050 | 0.8149 | 0.9336 |
| CCL20 | C-C motif chemokine 20 | P78556 | *CCL20* | 6364 | 0.019 | -0.169 | 0.214 | 0.8312 | 0.9492 |
| PRELP | Prolargin | P51888 | *PRELP* | 5549 | 0.007 | -0.061 | 0.075 | 0.8351 | 0.9506 |
| PI3 | Elafin | P19957 | *PI3* | 5266 | 0.012 | -0.105 | 0.129 | 0.8380 | 0.9508 |
| IgG Fc receptor II-b | Low affinity immunoglobulin gamma Fc region receptor II-b | P31994 | *FCGR2B* | 2213 | 0.015 | -0.142 | 0.167 | 0.8546 | 0.9592 |
| SCF | Stem cell factor | P21583 | *KITLG* | 4254 | -0.008 | -0.096 | 0.078 | 0.8560 | 0.9592 |
| RETN | Resistin | Q9HD89 | *RETN* | 56729 | -0.010 | -0.110 | 0.087 | 0.8560 | 0.9592 |
| FAS | Tumor necrosis factor receptor superfamily member 6 | P25445 | *FAS* | 355 | 0.006 | -0.061 | 0.075 | 0.8597 | 0.9592 |
| IL2-RA | Interleukin-2 receptor subunit alpha | P01589 | *IL2RA* | 3559 | 0.008 | -0.086 | 0.105 | 0.8604 | 0.9592 |
| PRSS27 | Serine protease 27 | Q9BQR3 | *PRSS27* | 83886 | 0.011 | -0.117 | 0.129 | 0.8623 | 0.9592 |
| BLM hydrolase | Bleomycin hydrolase | Q13867 | *BLMH* | 642 | 0.008 | -0.080 | 0.093 | 0.8644 | 0.9592 |
| CFHR5 | Complement factor H-related protein 5 | Q9BXR6 | *CFHR5* | 81494 | 0.008 | -0.083 | 0.099 | 0.8671 | 0.9592 |
| FGF-5 | Fibroblast growth factor 5 | Q8NF90 | *FGF5* | 2250 | 0.004 | -0.050 | 0.058 | 0.8734 | 0.9618 |
| KIM1 | Kidney Injury Molecule | Q96D42 | *HAVCR1* | 26762 | 0.012 | -0.138 | 0.166 | 0.8766 | 0.9618 |
| CCL14 | C-C motif chemokine 14 | Q16627 | *CCL14* | 6358 | -0.007 | -0.088 | 0.074 | 0.8803 | 0.9618 |
| TCN2 | Transcobalamin-2 | P20062 | *TCN2* | 6948 | -0.007 | -0.096 | 0.082 | 0.8803 | 0.9618 |
| CD93 | Complement component C1q receptor | Q9NPY3 | *CD93* | 22918 | 0.006 | -0.070 | 0.084 | 0.8835 | 0.9624 |
| IGFBP6 | Insulin-like growth factor-binding protein 6 | P24592 | *IGFBP6* | 3489 | 0.005 | -0.068 | 0.082 | 0.9022 | 0.9797 |
| PON3 | Paraoxonase | Q15166 | *PON3* | 5446 | 0.009 | -0.165 | 0.184 | 0.9139 | 0.9869 |
| TNFRSF10A | Tumor necrosis factor receptor superfamily member 10A | O00220 | *TNFRSF10A* | 8797 | -0.004 | -0.073 | 0.065 | 0.9144 | 0.9869 |
| CHIT1 | Chitotriosidase-1 | Q13231 | *CHIT1* | 1118 | 0.013 | -0.224 | 0.254 | 0.9198 | 0.9895 |
| EGFR | Epidermal growth factor receptor | P00533 | *EGFR* | 1956 | 0.002 | -0.044 | 0.048 | 0.9246 | 0.9895 |
| CCL19 | C-C motif chemokine 19 | Q99731 | *CCL19* | 6363 | 0.007 | -0.172 | 0.183 | 0.9262 | 0.9895 |
| Ep-CAM | Epithelial cell adhesion molecule | P16422 | *EPCAM* | 4072 | 0.010 | -0.232 | 0.254 | 0.9294 | 0.9895 |
| GAS6 | Growth arrest-specific protein 6 | Q14393 | *GAS6* | 2621 | 0.004 | -0.089 | 0.099 | 0.9308 | 0.9895 |
| LILRB2 | Leukocyte immunoglobulin-like receptor subfamily B member 2 | Q8N423 | *LILRB2* | 10288 | -0.003 | -0.073 | 0.069 | 0.9375 | 0.9906 |
| Gal-9 | Galectin-9 | O00182 | *LGALS9* | 3965 | -0.003 | -0.080 | 0.071 | 0.9409 | 0.9906 |
| CST3 | Cystatin-C | P01034 | *CST3* | 1471 | -0.003 | -0.087 | 0.082 | 0.9423 | 0.9906 |
| CCL3 | C-C motif chemokine 3 | P10147 | *CCL3* | 6348 | -0.005 | -0.155 | 0.141 | 0.9476 | 0.9906 |
| ITGB2 | Integrin beta-2 | P05107 | *ITGB2* | 3689 | 0.002 | -0.072 | 0.074 | 0.9482 | 0.9906 |
| TR-AP | Tartrate-resistant acid phosphatase type 5 | P13686 | *ACP5* | 54 | 0.003 | -0.086 | 0.096 | 0.9492 | 0.9906 |
| ANGPTL3 | Angiopoietin-related protein 3 | Q9Y5C1 | *ANGPTL3* | 27329 | -0.003 | -0.103 | 0.091 | 0.9514 | 0.9906 |
| PROC | Vitamin K-dependent protein C | P04070 | *PROC* | 5624 | 0.002 | -0.088 | 0.090 | 0.9562 | 0.9914 |
| COL1A1 | Collagen alpha-1(I) chain | P02452 | *COL1A1* | 1277 | 0.002 | -0.087 | 0.089 | 0.9578 | 0.9914 |
| IL-18R1 | Interleukin-18 receptor 1 | Q13478 | *IL18R1* | 8809 | 0.002 | -0.088 | 0.092 | 0.9653 | 0.9934 |
| PECAM-1 | Platelet endothelial cell adhesion molecule | P16284 | *PECAM1* | 5175 | -0.002 | -0.116 | 0.112 | 0.9664 | 0.9934 |
| GDF-15 | Growth/differentiation factor 15 | Q99988 | *GDF15* | 9518 | -0.001 | -0.092 | 0.087 | 0.9696 | 0.9934 |
| PRSS8 | Prostasin | Q16651 | *PRSS8* | 5652 | -0.002 | -0.097 | 0.096 | 0.9710 | 0.9934 |
| CCL4 | C-C motif chemokine 4 | P13236 | *CCL4* | 6351 | 0.002 | -0.140 | 0.142 | 0.9777 | 0.9974 |
| PLA2G7 | Platelet-activating factor acetylhydrolase | Q13093 | *PLA2G7* | 7941 | -0.001 | -0.053 | 0.052 | 0.9855 | 0.9989 |
| BMP-6 | Bone morphogenetic protein 6 | P22004 | *BMP6* | 654 | 0.001 | -0.175 | 0.178 | 0.9911 | 0.9989 |
| MMP-2 | Matrix metalloproteinase-2 | P08253 | *MMP2* | 4313 | 0.001 | -0.086 | 0.088 | 0.9927 | 0.9989 |
| PAI | Plasminogen activator inhibitor 1 | P05121 | *SERPINE1* | 5054 | 0.000 | -0.199 | 0.193 | 0.9960 | 0.9989 |
| NT-proBNP | N-terminal prohormone brain natriuretic peptide |  |  |  | -0.001 | -0.175 | 0.177 | 0.9960 | 0.9989 |
| CPB1 | Carboxypeptidase B | P15086 | *CPB1* | 1360 | 0.000 | -0.123 | 0.124 | 0.9965 | 0.9989 |
| IL-1 alpha | Interleukin-1 alpha | P01583 | *IL1A* | 3552 | 0.000 | -0.157 | 0.160 | 0.9989 | 0.9989 |

.
