# Supplementary material for: Longevity-associated BPIFB4 gene counteracts the inflammatory signaling
Source: Immun Ageing. 2024 Mar 12;21:19. doi: 10.1186/s12979-024-00424-5 (PMC10929107; doi:10.1186/s12979-024-00424-5)
Supplement: Supplementary file 5 — Supplementary Material 5 [file 12979_2024_424_MOESM5_ESM.docx]

|  | **Wilcoxon rank sum test** | | | |  | **QR:** Unadjusted | | |  | **QR:** Adjusted by sex | | |  | **QR:** Adjusted by LDL-C | | |  | **QR:** Adjusted by sex and LDL-C | | |  |
| --- | --- | --- | --- | --- | --- | --- | --- | --- | --- | --- | --- | --- | --- | --- | --- | --- | --- | --- | --- | --- | --- |
| **Protein symbol** | **Estimate** | **L95** | **U95** | **p-value** |  | **Beta** | **SE** | **p-value** |  | **Beta** | **SE** | **p-value** |  | **Beta** | **SE** | **p-value** |  | **Beta** | **SE** | **p-value** |  |
| TNFSF14 | **-0.21** | **-0.34** | **-0.07** | **0.0032** | ***** | **-0.17** | **0.08** | **0.0384** | ***** | **-0.19** | **0.08** | **0.0238** | ***** | **-0.19** | **0.08** | **0.0204** | ***** | **-0.21** | **0.08** | **0.0109** | ***** |
| PTPRS | **-0.06** | **-0.11** | **-0.02** | **0.0072** | ***** | **-0.07** | **0.03** | **0.0474** | ***** | -0.06 | 0.04 | 0.1212 |  | **-0.07** | **0.03** | **0.0378** | ***** | -0.06 | 0.04 | 0.1182 |  |
| CXCL11 | **-0.29** | **-0.49** | **-0.08** | **0.0072** | ***** | -0.32 | 0.16 | 0.0515 |  | -0.30 | 0.18 | 0.0854 |  | -0.31 | 0.17 | 0.0685 |  | -0.33 | 0.18 | 0.0630 |  |
| CXCL6 | **-0.27** | **-0.48** | **-0.07** | **0.0078** | ***** | -0.11 | 0.14 | 0.4223 |  | -0.13 | 0.14 | 0.3360 |  | -0.15 | 0.13 | 0.2488 |  | -0.16 | 0.13 | 0.2119 |  |
| ITGAM | **0.09** | **0.02** | **0.16** | **0.0081** | ***** | **0.13** | **0.05** | **0.0056** | ***** | **0.13** | **0.05** | **0.0042** | ***** | **0.10** | **0.05** | **0.0389** | ***** | **0.11** | **0.05** | **0.0292** | ***** |
| CD244 | **-0.12** | **-0.20** | **-0.03** | **0.0097** | ***** | **-0.11** | **0.04** | **0.0098** | ***** | **-0.12** | **0.04** | **0.0068** | ***** | **-0.12** | **0.04** | **0.0016** | ***** | **-0.14** | **0.04** | **0.0005** | ***** |
| IL-20RA | **-0.06** | **-0.11** | **-0.01** | **0.0111** | ***** | **-0.06** | **0.03** | **0.0200** | ***** | **-0.07** | **0.03** | **0.0060** | ***** | **-0.06** | **0.03** | **0.0267** | ***** | **-0.07** | **0.03** | **0.0073** | ***** |
| PRCP | **-0.09** | **-0.17** | **-0.02** | **0.0112** | ***** | **-0.09** | **0.03** | **0.0020** | ***** | **-0.12** | **0.03** | **0.0001** | ***** | **-0.10** | **0.03** | **0.0027** | ***** | **-0.12** | **0.03** | **0.0004** | ***** |
| REG3A | **-0.06** | **-0.11** | **-0.01** | **0.0127** | ***** | **-0.07** | **0.03** | **0.0117** | ***** | **-0.06** | **0.03** | **0.0410** | ***** | **-0.07** | **0.03** | **0.0111** | ***** | **-0.06** | **0.03** | **0.0243** | ***** |
| HSP 27 | **-0.13** | **-0.23** | **-0.03** | **0.0136** | ***** | -0.13 | 0.07 | 0.0778 |  | -0.13 | 0.07 | 0.0627 |  | -0.12 | 0.07 | 0.0998 |  | -0.13 | 0.07 | 0.0796 |  |
| CSF-1 | **-0.08** | **-0.14** | **-0.02** | **0.0137** | ***** | -0.03 | 0.03 | 0.3824 |  | -0.02 | 0.03 | 0.5114 |  | -0.02 | 0.03 | 0.4461 |  | -0.02 | 0.03 | 0.4769 |  |
| SLAMF7 | **-0.12** | **-0.21** | **-0.02** | **0.0147** | ***** | -0.03 | 0.05 | 0.5566 |  | -0.03 | 0.05 | 0.5987 |  | -0.04 | 0.06 | 0.4982 |  | -0.06 | 0.06 | 0.3335 |  |
| IDUA | **-0.25** | **-0.45** | **-0.05** | **0.0170** | ***** | -0.25 | 0.14 | 0.0739 |  | **-0.28** | **0.14** | **0.0462** | ***** | -0.25 | 0.14 | 0.0753 |  | **-0.28** | **0.14** | **0.0447** | ***** |
| Beta-NGF | **-0.06** | **-0.12** | **-0.01** | **0.0181** | ***** | -0.05 | 0.03 | 0.1522 |  | -0.05 | 0.03 | 0.1459 |  | -0.06 | 0.03 | 0.0978 |  | -0.06 | 0.03 | 0.0940 |  |
| NOTCH1 | **-0.06** | **-0.11** | **-0.01** | **0.0187** | ***** | **-0.08** | **0.03** | **0.0239** | ***** | **-0.08** | **0.04** | **0.0352** | ***** | **-0.07** | **0.03** | **0.0342** | ***** | **-0.08** | **0.04** | **0.0285** | ***** |
| IL-13 | **-0.05** | **-0.10** | **-0.01** | **0.0225** | ***** | **-0.04** | **0.02** | **0.0192** | ***** | **-0.05** | **0.02** | **0.0082** | ***** | **-0.05** | **0.02** | **0.0204** | ***** | **-0.05** | **0.02** | **0.0131** | ***** |
| CXCL1 | **-0.28** | **-0.52** | **-0.04** | **0.0227** | ***** | **-0.40** | **0.18** | **0.0263** | ***** | **-0.40** | **0.18** | **0.0240** | ***** | **-0.38** | **0.18** | **0.0371** | ***** | **-0.40** | **0.18** | **0.0263** | ***** |
| IL-2RB | **-0.06** | **-0.12** | **-0.01** | **0.0236** | ***** | -0.08 | 0.05 | 0.0744 |  | **-0.10** | **0.05** | **0.0371** | ***** | -0.07 | 0.05 | 0.1264 |  | -0.08 | 0.05 | 0.0829 |  |
| IFN-gamma | **-0.05** | **-0.09** | **-0.01** | **0.0240** | ***** | **-0.06** | **0.02** | **0.0239** | ***** | **-0.05** | **0.03** | **0.0336** | ***** | **-0.06** | **0.03** | **0.0358** | ***** | **-0.06** | **0.03** | **0.0262** | ***** |
| CXCL5 | **-0.36** | **-0.67** | **-0.05** | **0.0261** | ***** | -0.19 | 0.23 | 0.4192 |  | -0.26 | 0.21 | 0.2220 |  | -0.18 | 0.24 | 0.4552 |  | -0.25 | 0.22 | 0.2417 |  |
| TSLP | **-0.10** | **-0.20** | **-0.01** | **0.0264** | ***** | -0.11 | 0.06 | 0.0599 |  | -0.11 | 0.06 | 0.0675 |  | -0.10 | 0.05 | 0.0750 |  | -0.10 | 0.05 | 0.0765 |  |
| VCAM1 | **-0.08** | **-0.14** | **-0.01** | **0.0266** | ***** | -0.06 | 0.04 | 0.1151 |  | -0.06 | 0.04 | 0.1136 |  | -0.07 | 0.04 | 0.0900 |  | -0.07 | 0.04 | 0.1074 |  |
| IL-10RB | **-0.08** | **-0.14** | **-0.01** | **0.0268** | ***** | -0.06 | 0.03 | 0.0611 |  | **-0.08** | **0.04** | **0.0240** | ***** | **-0.07** | **0.03** | **0.0191** | ***** | **-0.08** | **0.04** | **0.0334** | ***** |
| SOD1 | **-0.11** | **-0.20** | **-0.01** | **0.0295** | ***** | -0.08 | 0.06 | 0.1811 |  | **-0.16** | **0.07** | **0.0144** | ***** | -0.09 | 0.06 | 0.1618 |  | **-0.15** | **0.07** | **0.0249** | ***** |
| LYVE1 | **-0.08** | **-0.15** | **-0.01** | **0.0331** | ***** | **-0.09** | **0.04** | **0.0336** | ***** | -0.07 | 0.04 | 0.0760 |  | **-0.09** | **0.04** | **0.0269** | ***** | -0.05 | 0.04 | 0.2362 |  |
| IL-6RA | **-0.10** | **-0.19** | **-0.01** | **0.0345** | ***** | -0.07 | 0.08 | 0.3846 |  | -0.07 | 0.08 | 0.3678 |  | -0.07 | 0.08 | 0.4033 |  | -0.07 | 0.08 | 0.3808 |  |
| TNFRSF9 | **-0.09** | **-0.18** | **-0.01** | **0.0352** | ***** | -0.07 | 0.05 | 0.1580 |  | -0.05 | 0.06 | 0.3842 |  | -0.08 | 0.05 | 0.1175 |  | -0.08 | 0.05 | 0.1207 |  |
| CASP-8 | **-0.22** | **-0.42** | **-0.01** | **0.0363** | ***** | -0.23 | 0.12 | 0.0528 |  | -0.22 | 0.11 | 0.0559 |  | **-0.24** | **0.12** | **0.0494** | ***** | **-0.23** | **0.11** | **0.0297** | ***** |
| FCGR3B | **-0.12** | **-0.23** | **-0.01** | **0.0399** | ***** | -0.09 | 0.06 | 0.1645 |  | -0.06 | 0.06 | 0.3482 |  | -0.08 | 0.07 | 0.2691 |  | -0.06 | 0.06 | 0.3210 |  |
| REG1A | **-0.11** | **-0.22** | **0.00** | **0.0424** | ***** | -0.13 | 0.07 | 0.0752 |  | -0.13 | 0.08 | 0.0758 |  | -0.13 | 0.07 | 0.0781 |  | -0.12 | 0.08 | 0.1260 |  |
| HGF | **-0.11** | **-0.21** | **0.00** | **0.0436** | ***** | -0.11 | 0.07 | 0.0973 |  | -0.11 | 0.07 | 0.1117 |  | -0.11 | 0.07 | 0.1177 |  | -0.11 | 0.07 | 0.1265 |  |
| TLT-2 | **-0.09** | **-0.19** | **0.00** | **0.0492** | ***** | -0.12 | 0.06 | 0.0622 |  | -0.10 | 0.06 | 0.0902 |  | -0.11 | 0.06 | 0.0910 |  | -0.11 | 0.06 | 0.0780 |  |
